# Catalytic Metal Ion–Substrate Coordination during Nonenzymatic RNA Primer Extension

**DOI:** 10.1021/jacs.4c00323

**Published:** 2024-04-05

**Authors:** Ziyuan Fang, Lydia T. Pazienza, Jian Zhang, Chun Pong Tam, Jack W. Szostak

**Affiliations:** † Department of Chemistry, Howard Hughes Medical Institute, The University of Chicago, Chicago, Illinois 60637, United States; ‡ Department of Chemistry and Chemical Biology, Harvard University, 12 Oxford Street, Cambridge, Massachusetts 02138, United States; § Department of Molecular Biology and Center for Computational and Integrative Biology, Howard Hughes Medical Institute, Massachusetts General Hospital, 185 Cambridge Street, Boston, Massachusetts 02114, United States

## Abstract

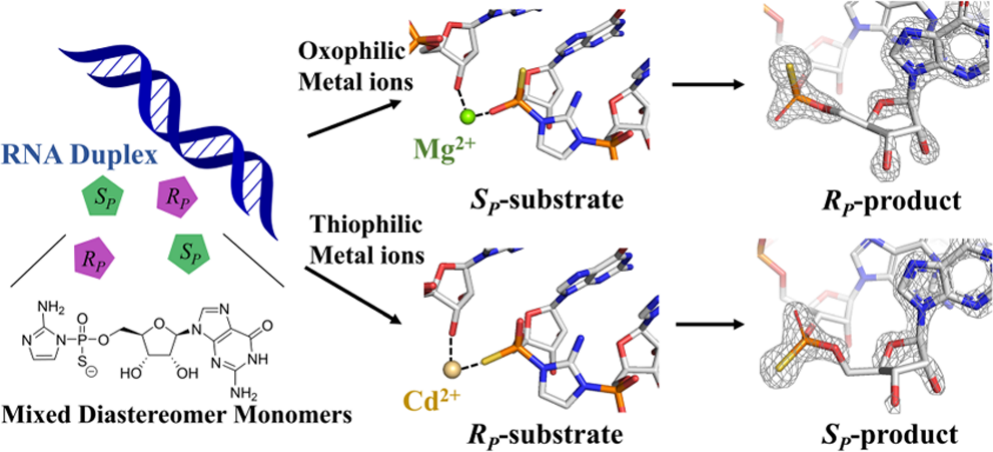

Nonenzymatic template-directed RNA copying requires catalysis by divalent metal ions. The primer extension reaction involves the attack of the primer 3′-hydroxyl on the adjacent phosphate of a 5′-5′-imidazolium-bridged dinucleotide substrate. However, the nature of the interaction of the catalytic metal ion with the reaction center remains unclear. To explore the coordination of the catalytic metal ion with the imidazolium-bridged dinucleotide substrate, we examined catalysis by oxophilic and thiophilic metal ions with both diastereomers of phosphorothioate-modified substrates. We show that Mg^2+^ and Cd^2+^ exhibit opposite preferences for the two phosphorothioate substrate diastereomers, indicating a stereospecific interaction of the divalent cation with one of the nonbridging phosphorus substituents. High-resolution X-ray crystal structures of the products of primer extension with phosphorothioate substrates reveal the absolute stereochemistry of this interaction and indicate that catalysis by Mg^2+^ involves inner-sphere coordination with the nonbridging phosphate oxygen in the pro-*S*_P_ position, while thiophilic cadmium ions interact with sulfur in the same position, as in one of the two phosphorothioate substrates. These results collectively suggest that during nonenzymatic RNA primer extension with a 5′-5′-imidazolium-bridged dinucleotide substrate the interaction of the catalytic Mg^2+^ ion with the pro-*S*_P_ oxygen of the reactive phosphate plays a crucial role in the metal-catalyzed S_N_2(P) reaction.

## Introduction

Nonenzymatic template-directed RNA copying is thought to have played a critical role in the emergence of the RNA world because it enabled the replication of genetic information prior to the evolution of ribozyme polymerases. Imidazole,^1^ 2-methyl-imidazole,^2,3^ and 2-amino-imidazole^4^-activated 5′-monophosphate ribonucleotides have been identified as potentially prebiotic substrates for template-directed RNA copying. However, achieving efficient nonenzymatic primer extension with these substrates demands concentrations of catalytic divalent metal ions ranging from tens to hundreds of millimolar.^5,6^ The *K*_M_ for Mg^2+^ in the nonenzymatic primer extension reaction is typically in the range of several 100 mM, indicating a very weak binding of the catalytic metal ion with the reaction center.^7^ Such high concentrations of divalent metal ions are not compatible with prebiotic vesicle systems based on fatty acids. Fatty acid vesicles initially aggregate and are then disrupted by high concentrations of divalent cations, such as Mg^2+^, leading to the release of vesicle contents. This effect of Mg^2+^ can be overcome either by chelating the Mg^2+^ with citrate^8^ or, to a lesser extent, through the addition of membrane-stabilizing compounds.^9^ We have previously shown that this incompatibility may be overcome through chelation of Mg^2+^ with citrate, suggesting that the catalytic Mg^2+^ ion interacts with the reaction center through at most three coordination sites.^10−12^ A better understanding of the coordination of the Mg^2+^ ion with functional groups in the reaction center could potentially guide the search for more prebiotically plausible chelators that preserve or even enhance the rate of primer extension while retaining compatibility with fatty acid-based vesicles.

While the functions of divalent metal cations are well studied in ribozyme- or enzyme-catalyzed nucleic acid polymerization reactions,^13−21^ their role in nonenzymatic RNA-templated copying remains unclear. The catalytic divalent cation has several potential functions in nonenzymatic RNA primer extension, including (1) stabilizing the primer/template duplex through electrostatic interaction;^22−24^ (2) facilitating deprotonation of the primer 3′-OH by providing an acceptor for the 3′-OH proton, through either inner- or outer-sphere coordination;^25,26^ (3) direct electrostatic stabilization of the deprotonated O3′ on the primer via inner-sphere interaction;^7,18^ and (4) dual-coordination of both O3′ and a nonbridging phosphate oxygen on the substrate to optimize the distance and angle of attack.^5,25,27^ Although high-resolution time-resolved crystal structures have helped to reveal the mechanism of the reaction,^28^ the catalytic metal ion has not been observed in the reaction center in these structures, presumably due to its weak binding.

**Figure 1:**
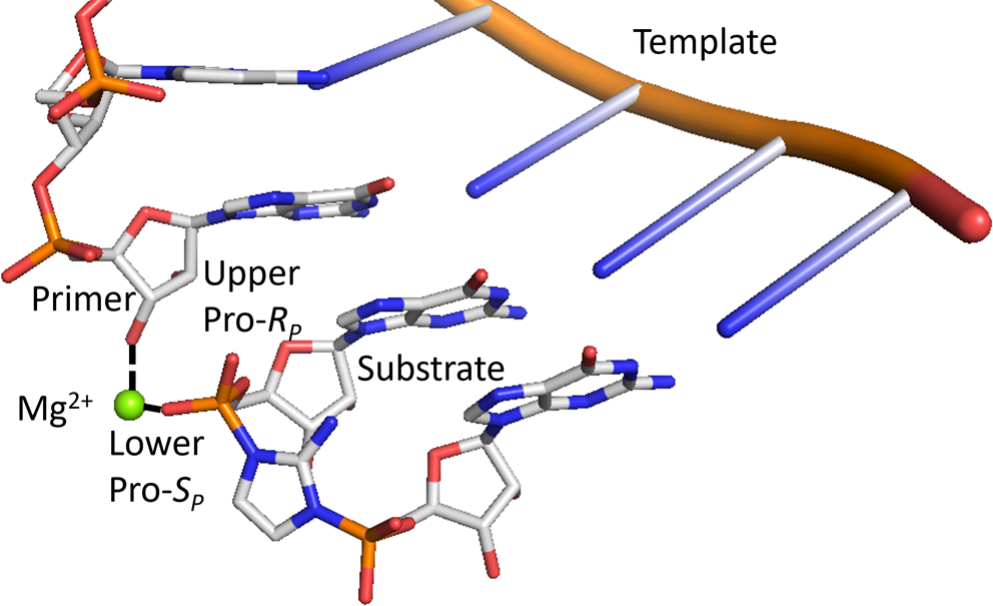
Model for coordination of the catalytic divalent ion in the nonenzymatic RNA primer extension reaction center. This model is based on MD simulations, suggesting that the Mg^2+^ ion coordinates with the “lower”, or pro-*S*_P_, oxygen.^29^

Molecular dynamics (MD) simulations suggest that the primer extension reaction is more favorable when the catalytic divalent ion forms inner-sphere contacts with the pro-*S*_P_ oxygen of the reactive phosphate of the substrate (the lower nonbridging oxygen in Figure 1).^29^ In our MD simulations, coordination of Mg^2+^ with the lower pro-*S*_P_ oxygen, as opposed to the upper pro-*R*_P_ oxygen, confers several advantages, including a more favorable angle for in-line attack and a slightly closer distance between the primer O3′ and the electrophilic P of the substrate. Additionally, this coordination geometry stabilizes the 3′-endo conformation of the ribose of the terminal primer nucleotide, which is critical for nonenzymatic primer extension.^26^ However, these MD simulation results do not account for important polarization and quantum mechanical effects and have not yet been subjected to experimental tests.

Here, we employed phosphorothioate-modified substrates to probe metal ion contacts with the reactive phosphate. Phosphorothioate modification of the substrate introduces a new chiral center on the phosphate by substituting one of the nonbridging oxygens with a sulfur atom. This modification has been widely applied in mechanistic studies of metal ion catalysis in nucleic acid research,^30,31^ including hammerhead cleavage,^32^ RNase P cleavage,^33^ spliceosome activity,^34^ and DNAzymes.^35^ With respect to nonenzymatic primer extension, if there is a selective coordination of the catalytic metal ion to one of the two nonbridging oxygens on the substrate phosphate, only one of the two phosphorothioate diastereomers would be expected to facilitate efficient primer extension depending on the metal ion used. Our initial kinetic studies with Mg^2+^ revealed a more than 10-fold difference in the maximal reaction rates of the two substrate diastereomers. Further exploration using both oxophilic and thiophilic metal ions revealed opposing preferences for the two phosphorothioate substrate diastereomers. Finally, we used X-ray crystallography to determine the absolute stereochemistry of the phosphorothioate RNA products. Oxophilic catalytic metal ions resulted in the *R*_P_ product, while thiophilic metal ions led to a reversed configuration. These findings are consistent with the results obtained from molecular dynamics simulations and contribute to our understanding of the involvement of catalytic divalent metal ions in nonenzymatic RNA primer extension.

## Results

### Mg^2+^-Catalyzed Nonenzymatic Primer Extension with Phosphorothioate Substrates

**Figure 2:**
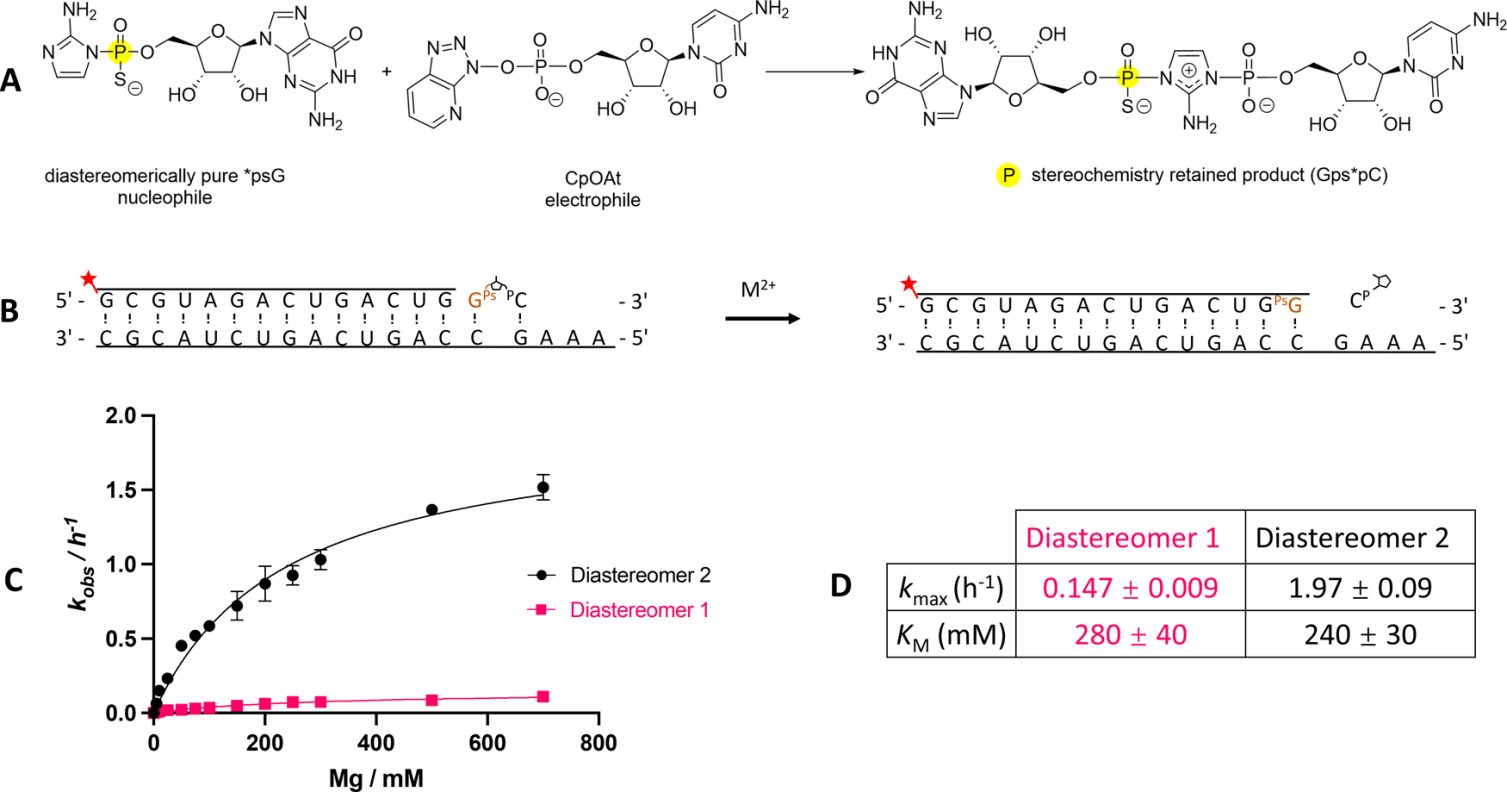
Kinetic study of magnesium ions for the primer extension reaction with two thiophosphoro-2-aminoimidazolium-bridged diastereomers. (A) Schematic representation of the in situ synthesis of thiophosphoro-2-aminoimidazolium-bridged dinucleotide (Gps*pC). (B) Schematic representation of nonenzymatic primer extension with Gps*pC. (C) Michaelis–Menten curve of Mg^2+^ for nonenzymatic primer extension with the two phosphorothioate substrate diastereomers. (D) Kinetic parameters for Mg^2+^ with the two diastereomers from fitting to the Michaelis–Menten equation. Primer extension reactions were performed with 1.7 μM primer, 2.6 μM template, 20 mM D1 or D2 *psG monomer, 20 mM CpOAt and 100 mM tris pH 8.0. Error bars indicate standard deviations of the mean, *n* = 3.

To investigate the coordination of the catalytic metal ion in the primer extension reaction center, we first synthesized 2-aminoimidazole-activated guanosine 5′-thiophosphate (*psG) and purified the two diastereomers. In the absence of structural evidence that would identify the (*R*_P_) and (*S*_P_) configurations, we refer to the two configurations as diastereomers 1 and 2 (D1 and D2). These labels correspond to their order of elution through preparative reverse phase HPLC. Since we were unable to purify sufficient diastereomerically pure activated bridged dinucleotides for our kinetic experiments, we opted to form the bridged dinucleotide (Gps*pC) in situ using the diastereomerically pure *psG monomer as the nucleophile for reaction with the highly reactive electrophile 1-hydroxy-7-azabenzotriazole activated cytidine (CpOAt) (Figure 2A).^36^ The formation of the bridged dinucleotide under these conditions is relatively rapid and retains the phosphorothioate stereochemistry due to exclusive attack of the 2AI moiety of *psG on the CpOAt phosphate.

We measured the rate of nonenzymatic primer extension as a function of Mg^2+^ concentration for both substrate phosphorothioate diastereomers. We used a Cy3-labeled 14-mer primer annealed to a 19-mer template to follow the nonenzymatic primer extension reaction (Figure 2B). We used a high concentration (20 mM) of in situ-formed phosphorothioate-modified bridged dinucleotide Gps*pC to saturate the primer/template complex with the substrate. Although the formation of the bridged dinucleotide intermediate and the subsequent primer extension reactions are sequential reactions in this model system, the initial step is relatively rapid, which allowed us to measure pseudo-first-order reaction rates for nonenzymatic primer extension. Extension rate constants were measured as a function of the Mg^2+^ concentration for the two phosphorothioate diastereomers, and the data were fitted to the Michaelis–Menten equation (Figure 2C,D). Notably, diastereomer 2 exhibited a 10-fold faster *k*_max_ compared to diastereomer 1. This difference could stem from either an intrinsic difference in reactivity unrelated to metal ion coordination or to a requirement for Mg^2+^ coordination to a specific nonbridging oxygen of the reactive phosphate. To distinguish between these possibilities, we proceeded to compare the reactivities of the two bridged substrates with oxophilic and thiophilic divalent metal ions.

### Oxophilic and Thiophilic Metal Ions Have Opposite Reactivity Preferences for the Two Diastereomers

Certain metal ions demonstrate a preference for binding to sulfur atoms over oxygen atoms, categorized as thiophilic metals. While this preference was traditionally considered related to the softness of the metal,^37,38^ recent research has uncovered a robust correlation between oxophilicity or thiophilicity and electronegativity.^39^ If the catalytic metal ion must coordinate with a specific nonbridging substituent on the reactive substrate phosphate, we expect to observe opposing preferences for the two diastereomeric substrates when substituting Mg^2+^ with a thiophilic metal cation, such as the divalent cadmium ion Cd^2+^. This kind of analysis has been used to investigate the role of metal ion catalysis in several self-cleaving ribozymes, notably the Varkud satellite (VS) ribozyme where very large thio effects and metal-rescue effects were observed.^40^ Therefore, we measured the reaction rate in the absence of metal ions and in the presence of oxophilic metal ions (magnesium and manganese) and thiophilic metal ions (cadmium). The primer extension reactions were conducted with the two phosphorothioate diastereomers of the Gps*pC-bridged dinucleotides, which were purified by HPLC after synthesis from diastereomerically pure *psG monomer and CpOAt. This avoided precipitation due to the presence of excess monomer. The reaction conditions were also adjusted to minimize precipitation in the presence of Cd^2+^ by decreasing the pH to 7.0 and utilizing only 10 mM of purified Gps*pC. We also measured reaction rates with all three metals with the native 2AI-bridged dinucleotide (Gp*pC) as a control (Table 1).

**Table 1:** Observed Primer Extension Rates and Thio Effect for Different Metal Ions with the Two Phosphorothioate Diastereomers^a^

	native Gp*pC	diastereomer 1		diastereomer 2	
metal ion	*k*_obs_ (h^–1^)	*k*_obs_ (h^–1^)	*k*_O_/*k*_S_	*k*_obs_ (h^–1^)	*k*_O_/*k*_S_
none	0.0035(2)	0.00033(2)	11(1)	0.00068(3)	5.1(5)
Mg^2+^	1.02(6)	0.0039(2)	260(30)	0.126(5)	8.1(8)
Mn^2+^	7.4(3)	0.29(4)	26(5)	0.62(3)	12(1)
Cd^2+^	0.37(2)	1.28(4)	0.29(2)	0.138(5)	2.7(2)

a Primer extension reactions were performed 1.7 μM primer, 2.6 μM template, 10 mM bridged dinucleotide, 50 mM metal chloride, and 100 mM tris pH 7.0. The last bracketed digit indicates standard deviations of the mean, *n* = 3.

The two phosphorothioate substrate diastereomers exhibit similarly low reaction rates in the absence of divalent metal ions, indicating that the two diastereomers do not differ greatly in their intrinsic reactivity. As shown above, D1 exhibits a thio effect (*k*_O_/*k*_S_) of 260-fold compared to 8-fold for D2 and is at least 10-fold less reactive than D2 in the presence of the oxophilic metal ion Mg^2+^. Mn^2+^ also leads to higher reactivity with D2 compared to that of D1, though the difference is much smaller than that with Mg^2+^, consistent with the fact that Mn^2+^ is less oxophilic than Mg^2+^. Interestingly, in the presence of thiophilic ion Cd^2+^, D1 reacts 10-fold faster than D2. This reversed substrate selectivity of the thiophilic Cd^2+^ supports the hypothesis that the catalytic Mg^2+^ ion exhibits a specific inner-sphere interaction with one of the nonbridging phosphate oxygens of the reactive phosphate of the bridged dinucleotide during primer extension. We note that the rate of Cd^2+^-catalyzed primer extension is 3.5-fold greater with the D1 phosphorothioate substrate than with the native substrate, suggesting that when Cd^2+^ interacts with a correctly positioned sulfur atom, it is such an effective catalyst that it can overcome the intrinsic thio effect of 11-fold. As a result, Cd^2+^ confers a net rate enhancement of almost 4000-fold on the D1 substrate versus 200-fold on the D2 substrate and 100-fold on the native substrate. However, without structural information, whether this coordination was with the upper or lower nonbridging oxygen remained unknown.

### Product Structures of Mg^2+^-Catalyzed Primer Extension with Phosphorothioate Substrates

**Figure 3:**
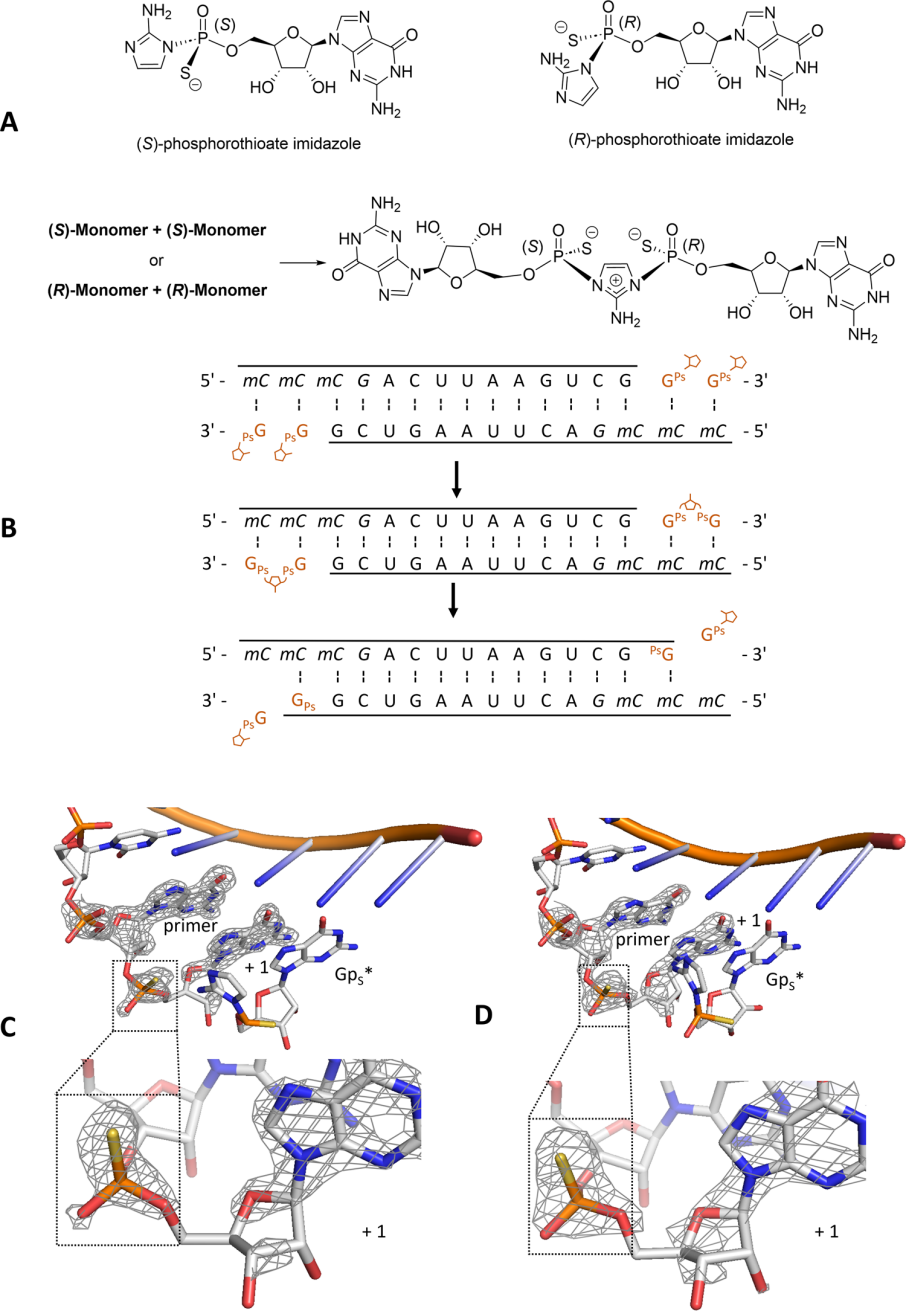
Structures of the product of Mg^2+^-catalyzed primer extension with two diastereomeric monomers. (A) Schematic representation of the thiophosphoro-2-aminoimidazolium-bridged dinucleotide formed by the same diastereomer monomer. (B) Schematic representation of the nonenzymatic primer extension reactions with thiophosphoryl-monomers. (C) Product of Mg^2+^-catalyzed primer extension with diastereomer 1 (PDB: 7U89). (D) Product of Mg^2+^-catalyzed primer extension with diastereomer 2 (PDB: 7U8A). (Meshes indicate the composite omits 2DF_o_-mF_c_ maps contoured at 2.0 σ).

We investigated the absolute stereochemistry of the product of Mg^2+^-catalyzed primer extension with phosphorothioate substrates by X-ray crystallography. We employed a partially self-complementary RNA 14-mer to establish a primer/template complex (Figure 3B). This complex served as the basis for acquiring atomic-resolution crystal structures in our previous investigation of the mechanism of nonenzymatic RNA primer extension.^28,41,42^ Given that both the +1 and +2 positions on the template are locked 5-Me-C, only *psG was utilized as the substrate so that the Gps*psG bridged dimer was formed in situ either on the template or in solution followed by binding to the template. Although we used the two different diastereomeric *psG monomers, they ultimately result in the same (R, S)-Gps*psG substrate due to the inversion of chirality at one phosphorus center during the formation of the bridged dinucleotide. Consequently, the two sides of the bridged dinucleotide exhibit opposite phosphate chirality (Figure 3A). This substrate allows for two possibilities when binding to the primer/template complex: (S)-phosphate at the +1 and (R)-phosphate at the +2 position, or (R)-phosphate at the +1 and (S)-phosphate at the +2 position. Based on the results described above, we suspected that only one of the two potential binding modes would lead to primer extension, and the product of primer extension would therefore exhibit the same chirality on the +1 position irrespective of the use of D1 or D2 as the starting monomer.

The resulting primer extension products were crystallized directly without further purification, and we were able to determine the structures of both product duplexes (Figure 3C). Both products display stronger electron density at the upper position (upper sulfur) and weaker density at the lower position (lower oxygen). Therefore, both products have the same chirality (*R*_P_) on the +1 phosphorothioate, as expected. We quantitatively confirmed the configurations of these structures through phenix occupancy refinement (Table S1)^43,44^ and B factor constancy validation (Figure S1).^45^ This result is consistent with the prediction that the bridged dinucleotide Gps*psG exhibits different phosphate chirality on the two sides, and only one of its two potential binding modes is conducive to the formation of primer extension products in the presence of Mg^2+^. Furthermore, the *R*_P_ chirality on the product (upper sulfur and lower oxygen) indicates that primer extension is possible only when the phosphate with lower oxygen and upper sulfur is bound at the +1 position. Given the oxophilic nature of Mg^2+^ ions, the lower oxygen content (pro-*S*_P_) plays a critical role in Mg^2+^ catalysis. Our findings are consistent with the conclusions drawn from MD simulations.^29^

We extended the above experiment by using a 13-mer primer, one nucleotide shorter at its 3′-end, so that the product of primer extension had added two phosphorothioate nucleotides. The structures consistently displayed *R*_P_ products at both the +1 and +2 positions, regardless of whether D1 or D2 was used as a substrate (Figure S2), further supporting the stereospecific coordination of Mg^2+^ with the pro-*S*_P_ oxygen.

### Product Structures of Cd^2+^-Catalyzed Primer Extension with Phosphorothioate Substrates

The above results prompted us to investigate whether a thiophilic metal ion would lead to a reversed configuration of the phosphorothioate product. Since both D1 and D2 yielded the same product with Mg^2+^, we avoided the tedious separation of the diastereomeric monomers and simply employed the mixture of diastereomeric phosphorothioate monomers for in situ formation of Gps*psG. However, the Cd^2+^-catalyzed primer extension reaction did not yield sufficient product to proceed directly to crystallization without the removal of unreacted primer. Consequently, we purified the product via HPLC and conducted a parallel experiment using Mg^2+^ as a control.

**Figure 4:**
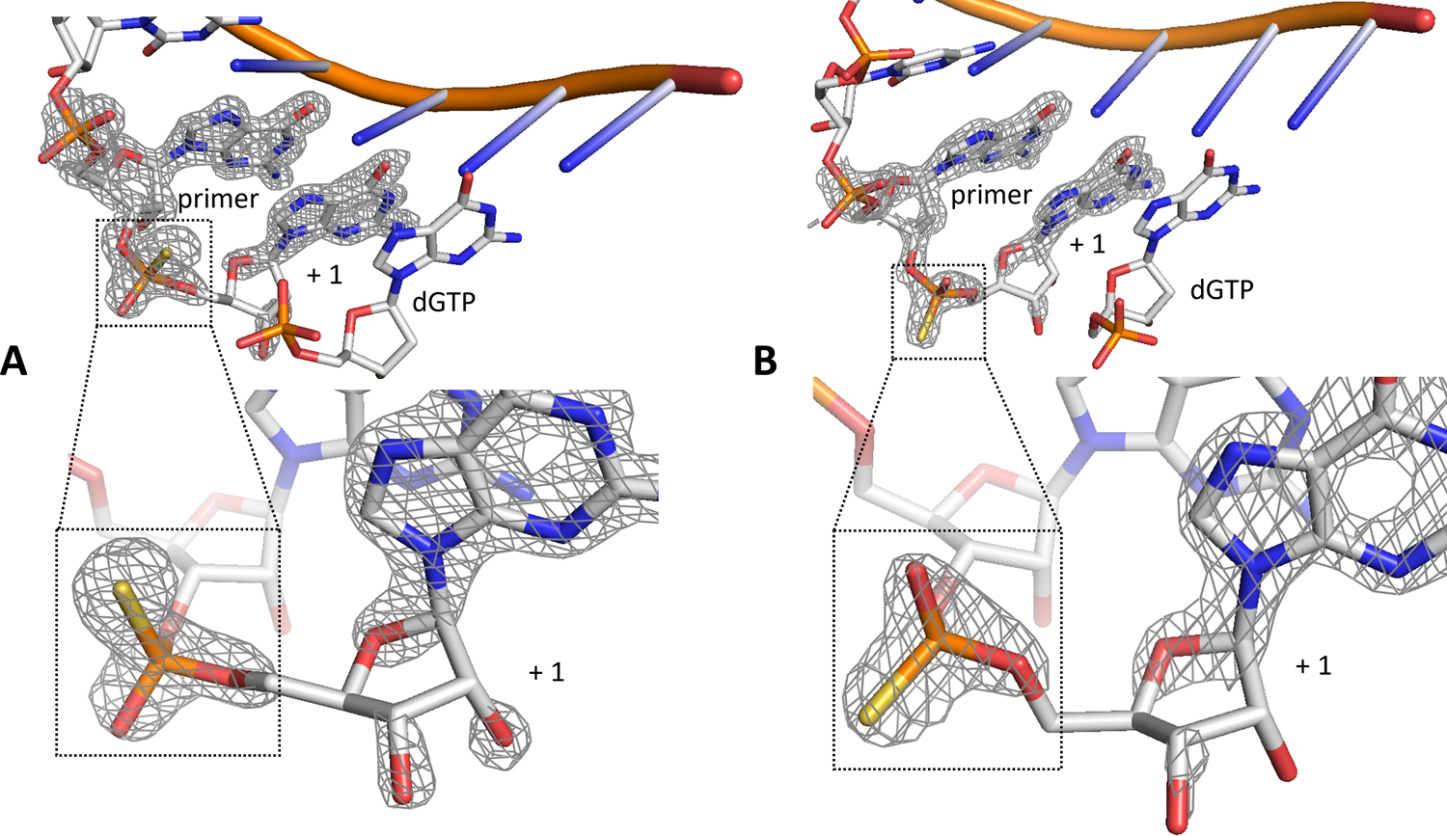
Structure of the products of Mg^2+^- or Cd^2+^-catalyzed primer extension with mixed diastereomeric monomers. (A) Product structure of the Mg^2+^-catalyzed primer extension reaction with mixed *psG diastereomers (PDB: 8VAW). (B) Product structure of the corresponding Cd^2+^-catalyzed primer extension reaction (PDB: 8VAX). (Meshes indicate the composite omit 2DF_o_-mF_c_ maps contoured at 2.0 σ).

The Mg^2+^-catalyzed product once again displays *R*_P_ chirality for the +1 phosphorothioate (Figure 4A). In contrast, when the reaction is catalyzed by thiophilic Cd^2+^ ions, the product exhibits a weaker upper density (upper oxygen) and stronger lower density (lower sulfur), indicating the *S*_P_ chirality (Figure 4B). These configurations were quantitatively validated as described above (Table S1 and Figure S1). This stereochemically inverted product is consistent with our kinetic experiments and with Cd^2+^ coordination with sulfur on the substrate rather than with oxygen. These findings further substantiate our hypothesis that the catalytic metal ion must engage in an inner-sphere interaction with the lower nonbridging oxygen or sulfur of the reactive phosphate.

## Discussion

Gaining insight into the coordination of the catalytic divalent metal ion with the substrate is an important aspect of understanding the overall mechanism of nonenzymatic RNA primer extension. A better mechanistic understanding may lead to the identification of reaction conditions that maintain or improve primer extension rates, potentially facilitating cycles of nonenzymatic replication. To address the coordination of the catalytic metal ion within the reaction center, we employed phosphorothioate substrates, utilizing both reaction kinetics and X-ray crystallography to define the metal ion–substrate interaction.

A significant problem in pursuing metal ion rescue experiments with 2AI-activated phosphorothioate substrates is that producing 100% pure diastereomers is impractical due to the reversible nature of the reaction between activated monomers that generate bridged dinucleotides. Even in the absence of metal ions, diastereomerically pure activated phosphorothioate monomers react with each other to form bridged dinucleotides, resulting in inversion of chirality on one of the phosphates (Figure 3A). These bridged dinucleotides can then either react with free 2AI or simply hydrolyze to generate monomers with the opposite diastereomeric configuration. We observed both of these unwanted side products by HPLC after lyophilization (Figure S3). Notably, after several freeze–thaw cycles, a substantial amount of the alternate monomer diastereomer (∼5%) was detected. Considering this unavoidable substrate impurity, the actual difference between the rate constants for the two diastereomers may be even larger than that seen experimentally.

Our kinetic experiments revealed distinct preferences of Mg^2+^ and Cd^2+^ for the two phosphorothioate substrate diastereomers. Substituting the coordinating oxygen with sulfur typically leads to a decrease in the reaction rate in Mg^2+^-catalyzed RNA reactions such as for self-splicing introns or RNase P cleavage.^46^ The recovery of the diminished reaction rate with a thiophilic metal ion (Cd^2+^ in our case) is defined as “metal ion rescue”. This effect can be quantified by the following equation^47,48^
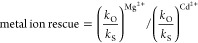
where *k*_O_ is the *k*_obs_ for the native substrate and *k*_S_ is the *k*_obs_ for the phosphorothioate substrate. Accordingly, the metal ion rescue effect for D1 is almost 1000-fold, while for D2, it is only a factor of 3. The significantly stronger metal ion rescue effect observed for the Mg^2+^-disfavored substrate D1 suggests that the sulfur substitution on D1 replaces the crucial metal coordination nonbridging oxygen position. Consequently, the observed opposite diastereoselectivity with oxophilic and thiophilic metal ions strongly supports the hypothesis that the catalytic metal ion must bind to one of the nonbridging oxygen (or sulfur) atoms of the reactive phosphate.

**Figure 5:**
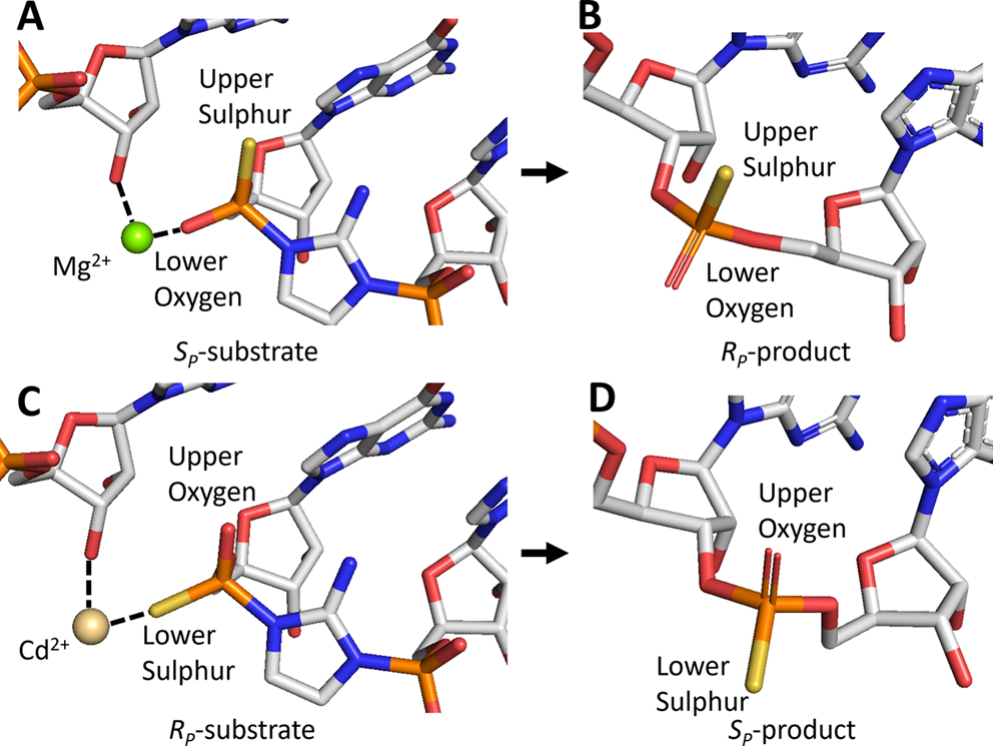
Presumed primer extension reaction mechanism with phosphorothioate substrates. (A) Mg^2+^ coordinates to the lower oxygen on *S*_P_-substrate. (B) Mg^2+^ catalysis leads to *R*_P_-product. (C) Cd^2+^ coordinates to the lower sulfur on *R*_P_-substrate. (D) Cd^2+^ catalysis leads to *S*_P_-product (parallel-eye stereo pairs are present in Figure S4).

We have so far been unable to directly determine the absolute stereochemistry of the two diastereomeric-activated phosphorothioate monomers or of the corresponding bridged dinucleotide substrates. According to the literature, the (*R*_P_)-diastereomer of phosphorothioate nucleoside triphosphates tends to elute first on reversed-phase HPLC.^49,50^ If the same trend holds for thiophosphoryl-2-aminoimidazolides, then D1 is likely the (*R*_P_)-diastereomer, in which the lower oxygen is substituted by sulfur (Figure 5C). Conversely, the Mg^2+^-favored substrate D2 is likely to be the (*S*_P_)-diastereomer, with the upper oxygen being substituted by sulfur (Figure 5A). The observed greater reactivity of the D2 substrate with Mg^2+^ ions, together with the results of MD simulations, suggested that inner-sphere coordination with the lower pro-*S*_P_ oxygen atom favors reactivity.

The above speculation concerning the absolute stereochemistry of the favored phosphorothioate substrates is robustly supported by the product structures, as determined by X-ray crystallography. Mg^2+^ consistently leads to *R*_P_ products, regardless of the stereochemistry of the substrate monomer (Figure 5B). Conversely, primer extension products catalyzed by Cd^2+^ exhibit the opposite stereochemistry (*S*_P_) (Figure 5D). Previous work supports an S_N_2-like reaction mechanism in phosphorimidazolide hydrolysis,^51^ so the stereospecific products we observed support a similar mechanism in primer extension. There are considerable mechanistic similarities between primer extension and the ribozyme-catalyzed cleavage of RNA by transphosphorylation, which involves deprotonation of the 2′-hydroxyl nucleophile, stabilization of the in-line geometry for nucleophilic attack, electrophilic phosphate activation, and stabilization of the leaving group,^52^ which provide a helpful context for interpreting the mechanism of metal ion-catalyzed primer extension. As an example, metal ions play a similar role in the HDV ribozyme by coordinating to a nonbridging phosphoryl oxygen and activating the nucleophile.^53^ If primer extension proceeded through a fully dissociative mechanism, then the products would exhibit mixed stereochemistry, which we did not observe. Assuming an S_N_2-like mechanism (Figure S5), reactant substrate chirality can be deduced from that of the products. Therefore, Mg^2+^ catalyzes the reaction when the *S*_P_ substrate binds at the +1 position (Figure 5A), while Cd^2+^ catalyzes the reaction when the *R*_P_ substrate binds at the +1 position (Figure 5C). Given the nature of oxophilic and thiophilic metal ions, the presence of oxygen or sulfur at the pro-*S*_P_ position of the reactive substrate phosphate is crucial for Mg^2+^ or Cd^2+^ coordination, respectively.

## Conclusions

Our determination of the nature of coordination of the catalytic metal ion with the reactive phosphate of the substrate sets the stage for future investigations of the mechanism of nonenzymatic primer extension. A key remaining unknown is whether the catalytic metal ion is directly inner-sphere-coordinated with the primer 3′-OH or whether that interaction is a more indirect outer-sphere coordination. The nature of the reaction pathway also remains unclear, i.e., does the catalytic metal ion first coordinate with the reactive phosphate, followed by outer-sphere coordination and then inner-sphere coordination with the 3′-hydroxyl, leading to deprotonation and then attack of the alkoxide on the phosphate? At what point during the chemical step of the reaction does the metal ion dissociates from primer O3′? Finally, we suggest that knowing the correct geometry of the interaction of the catalytic metal ion with the reaction center may facilitate the design or discovery of small molecule chelators or peptides that could help to catalyze primer extension by stabilizing the weak interaction of the catalytic metal ion with the reaction center.

## Supporting Information Available

Materials and methods; supporting discussion; and supporting Figures and Tables (PDF)
