# Supplementary material for: Catalytic Metal Ion–Substrate Coordination during Nonenzymatic RNA Primer Extension
Source: J Am Chem Soc. 2024 Apr 5;146(15):10632–9. doi: 10.1021/jacs.4c00323 (PMC11027144; doi:10.1021/jacs.4c00323)
Supplement: Supplementary file 1 — ja4c00323_si_001.pdf [file ja4c00323_si_001.pdf]

Supplementary Information

Catalytic Metal Ion - Substrate Coordination during Nonenzymatic RNA Primer Extension

Ziyuan Fang[1†], Lydia T. Pazienza[2,3†], Jian Zhang[1], Chun Pong Tam[2,3] and Jack W. Szostak[1*]

[1] Howard Hughes Medical Institute, Department of Chemistry, The University of Chicago, Chicago, Illinois 60637, USA.

[2] Department of Chemistry and Chemical Biology, Harvard University, 12 Oxford Street, Cambridge, Massachusetts 02138, USA.

[3] Howard Hughes Medical Institute, Department of Molecular Biology and Center for Computational and Integrative Biology, Massachusetts General Hospital, 185 Cambridge Street, Boston, Massachusetts 02114, USA.

[†]Z.F. and L.P. contributed equally to this paper.

*To whom correspondence should be addressed; Email: jwszostak@uchicago.edu.

Table of contents

Supplementary Discussion S2

Materials and Methods S3

Supplementary Figures and Tables S9

References S26

Supplementary Discussion

The observed K_m values for Mg^{2+} for primer extension reactions with the two substrate phosphorothioate diastereomers were not significantly different (Figure 1C-D), even though the reaction rates were very different. We suggest that a plausible explanation for the apparently similar Mg^{2+} binding is that the residual activity observed with the poorly reactive diastereomer could be a result of the presence of a small amount of the other diastereomer. As elaborated in the main discussion section, the persistence of some residual opposite diastereomer post-purification and lyophilization is a known and unavoidable phenomenon (Figure S3).

While the electron density maps of the products of primer extension with phosphorothioate substrates offer compelling visual evidence of stereospecific product formation, they lack quantitative precision. To address this, we employed two analytical methods to derive quantitative insights into the enantiomeric excess of the product phosphorothioates. Our initial approach involved phenix occupancy refinement[1, 2] of two alternative models for +1 R_P and S_P product configurations. In these models all corresponding atoms were at identical locations except for the sulfur atoms. The coordinates for the sulfur atoms were calculated using known bond lengths for P-S bonds. The occupancies for these two alternative conformers were obtained by optimization of the occupancy parameters during the refinement process (Table S1). All Mg^{2+} catalyzed products exhibited over 97% R_P configuration, whereas the cadmium-catalyzed products exhibited less than 6% R_P product. This quantitative analysis confirms of the chirality of the products.

To further confirm the accuracy of the occupancy refinement results, we examined the constancy in B-factors across different population ratios, relying on the rigid-body assumption.[3] This approach is rooted in the expectation that B-factors should be erroneously larger than those of neighboring atoms when the population occupancy is over or underestimated. The B values were computed post-refinement with pre-fixed occupancies, and constancy was evaluated using the following index:

$$\text{Constancy Index} = \sqrt{(\mathrm{B_S}(R_P) - \mathrm{B_S}(S_P))^2 + (\mathrm{B_O}(R_P) - \mathrm{B_O}(S_P))^2}$$

B(S_P) and B(R_P) denote the B-factors for the two diastereomers, with subscripts S and O indicating sulfur and oxygen atoms, respectively. The results of this analysis closely aligned with the occupancies calculated during the structure refinement.

Materials and Methods

General information

All chemicals were purchased from Sigma-Aldrich (St. Louis, MO) and used without purification unless otherwise noted. Phosphoramidites and reagents used for solid-phase RNA synthesis were purchased from ChemGenes (Wilmington, MA) and Glen Research (Sterling, MA). Deuterated solvents were purchased from Cambridge Isotope Laboratories (Tewksbury, MA) and Thermal Scientific.

Preparatory-scale high performance liquid chromatography (HPLC) was carried out on a Teledyne Isco ACCQprep HP150 system, equipped with either of two columns. We used a Waters Atlantis T3 OBD C18 Column (100Å, 19 mm x 250 mm, 5μm) for the separation of the diastereomers of activated phosphorothioate monomers. We used an Agilent ZORBAX Eclipse XDB C18 column (80Å, 21.2 mm x 250 mm, 7 μm) for purifications of intermediate compounds, CpOAt and bridged dinucleotides. Analytical HPLC was carried out on a Shimadzu LC-20 system equipped with a Waters Atlantis T3 C18 Column (100Å, 4.6 mm x 150 mm, 3μm). The purity of all synthesized products was determined either by NMR or high-resolution mass spectrometry (HRMS). ^{1}H, ^{13}C and ^{31}P spectra were acquired on a Varian Oxford AS-400 NMR, Bruker Ascend™ 400 or Bruker Ultrashield™ 500 Plus spectrometer (400 or 500 MHz for ^{1}H, 101 or 126 MHz for ^{13}C, 162 MHz for ^{31}P) at 25 °C. HRMS was carried out on Agilent 6520 Q-TOF LC-MS with an electrospray ionization (ESI) source.

Synthesis of guanosine thiophosphoro-2-aminoimidazolides (*psG)

The 2-aminoimidazolides were synthesized in a two-stage procedure, from the 2-methyl imidazolides.

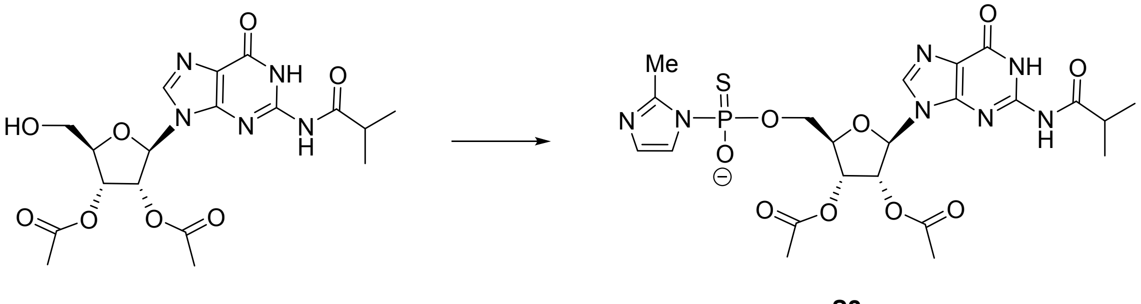

S1 S2

[1.1] *N*2-isobutyryl-2′,3′-diacetylguanosine **S1** (1.75 g, 4 mmol) was charged into a flask with positive pressure argon in a Schlenk line setup. Anhydrous acetonitrile (15 mL) was used to dissolve the white solid completely. 2,6 lutidine (2.3 mL, 20 mmol) was subsequently added to the mixture, followed by the slow, drop-wise addition of $PSCl_3$ (808 µL, 8mmol, caution: TOXIC). The resulting mixture was stirred at room temperature for 3 hours until complete consumption of starting substrate *N*2-isobutyryl-2′,3′-diacetylguanosine (traced by silica gel TLC, MeOH/EtOAc = 1/40). [1.2] 2-methyl imidazole (3.94 g, 48 mmol) was then added in one portion and the resulting mixture was stirred at room temperature for 20 min. The flask was then placed into an ice bath and 40 mL TEAB (100 mM, pH 7.5) was added. The resulting mixture was stirred at 0 ºC for 1 hour (there was only a small amount of the desired product at that time point as seen by HPLC). The resulting mixture was placed in a freezer (-20 ºC) overnight and the target product **S2**, with mass m/z 596 (negative mode), was purified by C18 reverse chromatography.

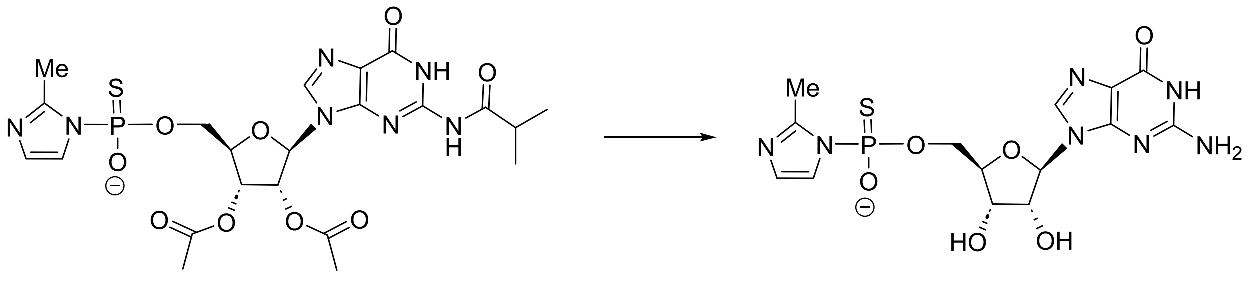

S2 **S3**

(diastereomeric mixture)

Compound **S2** prepared above was transferred to a heavy-walled round bottom pressure flask with MeOH (2 mL) and 20 mL $NH_3.H_2O$ was added, then the flask was tightly sealed. The flask was placed into an oil bath (65 ºC) and stirred for 3 hours. After cooling down to room temperature, the mixture was vacuum evaporated, dissolved in 16 mL TEAB, and purified by C18 reverse chromatography.

^{1}H NMR (400 MHz, D_2O) δ 7.77 and 7.71 (s, 1H), 7.12-7.10 (m, 1H), 6.58-6.5 (m, 1H), 5.70-5.68 (m, 1H), 4.54 and 4.50 (t, J = 5.6 Hz, 1H), 4.25-4.22 (m, 1H), 4.11-4.08 (m, 1H), 3.92-3.84 (m, 1H) (Figure S6); ^{13}C NMR **(**101 MHz, D_2O**)** δ 169.54 (169.47), 162.99 (162.93), 154.24 (154.21), 150.60 (150.58, 150.56, 150.54), 137.96 (137.88), 128.04 (127.93), 124.92 (124.84), 120.1, 88.97 (88.94), 85.71 (85.61, 85.58, 85.49), 76.10 (76.02), 73.28 (73.12), 68.41 (68.35, 68.30, 68.24), 48.8 ($TEAB^+$), 17.07 (17.04), 11.1 ($TEAB^+$) (Figure S7)**.**

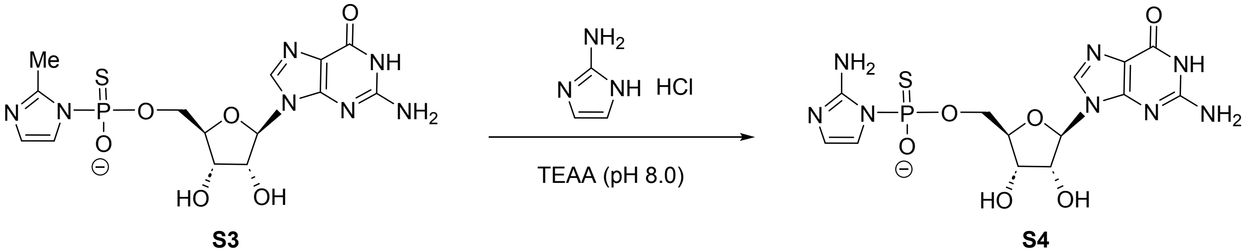

S3 **S4**

(diastereomeric mixture)

2-Methylphosphorimidazolide **S3** (230 mg, 0.52 mmol), and 2-aminoimidazole hydrochloride (1.24 g, 52 mmol) were dissolved in 4 mL TEAA (2M, pH 7.0), and the resulting mixture (pH <

7.0) was adjusted to pH 8.0 with triethylamine and stirred at room temperature. The reaction was monitored by HPLC. Upon completion of the reaction (>98% yield), the racemic product **S4** was purified by C18 reverse chromatography. ^{1}HNMR of **S4** (diastereomeric mixture) (400 MHz, D_2O) δ 7.88-7.86 (m, 2H), 7.76-7.73 (m, 2H), 6.41-6.39 (m, 2H), 5.81-5.79 (m, 2H), 4.63-4.58 (m, 2H), 4.32-4.27 (m, 2H), 4.21 (s, 2H), 4.09-3.91 (m, 4H) (Figure S8). ^{31}P NMR of the mixture (162 MHz, D_2O) δ 46.75, 46.55 (Figure S9).

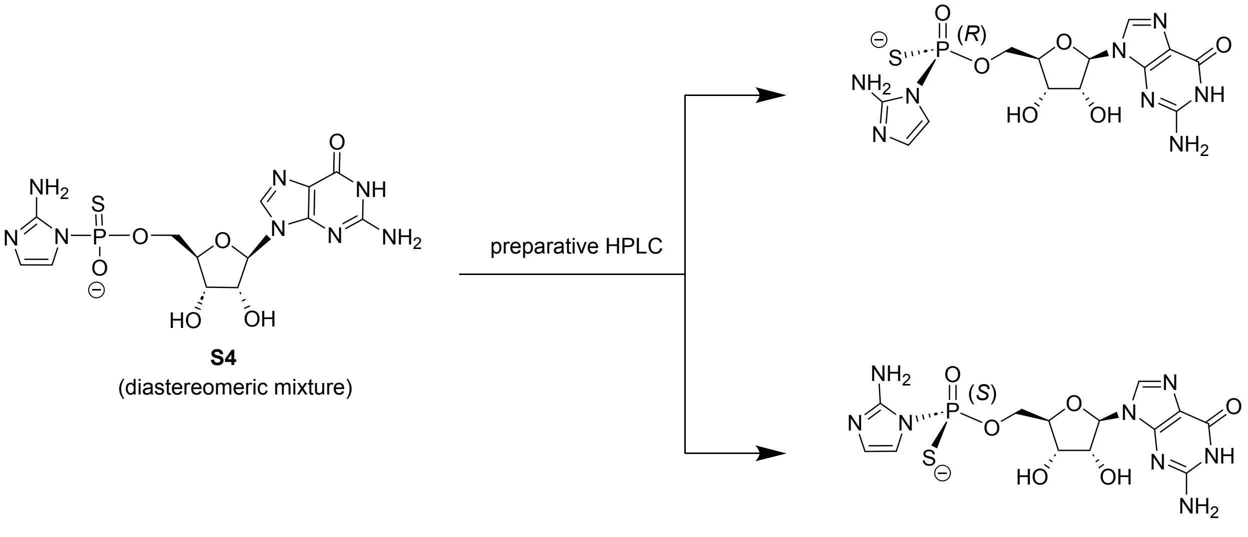

(*S*)-phosphorothioate imidazole, **(*S*)-7**

The two diastereomers **(*R*)-7** and **(*S*)-7** were separated by preparative HPLC (Atlantis™ T3 Prep OBD™, 5 µm, 19×250 mm, part No. 186004026, serial No. 02073307411402). Separation of the diastereomers often took 2-3 purification cycles to completely remove the opposing diastereomer from the samples. The purity of the separated fraction was analyzed by analytical HPLC (Atlantis™ T3 3µm, 4.6×150 mm, part No. 186003729, serial No. 02053127214020). Since we have not identified which diastereomer is the (*R*) or (*S*) diastereomer, the diastereomer that has a shorter retention time is called diastereomer 1 (D1) and the one with a longer retention time is called diastereomer 2 (D2).

^{1}H NMR of **diastereomer 1** (400 MHz, 10% D_2O in H_2O) δ 7.92 (s, 1H), 6.73 (s, 1H), 6.40 (s, 1H), 5.82 (d, J = 5.6 Hz, 1H), 4.40-4.37 (m, 1H), 4.23-4.22 (m, 1H), 4.14-4.08 (m, 2H), 4.03-3.98 (m, 1H) (Figure S10). ^{31}P NMR of **diastereomer 1** (162 MHz, 10% D_2O in H_2O) δ 46.93 (Figure S11). ^{13}C NMR of **diastereomer 1 (**101 MHz, D_2O) δ 218.0 (acetone), 162.2, 156.9, 154.3, 153.1, 140.1, 124.0, 119.1, 118.71 (118.64), 89.8, 86.00 (85.90), 76.0, 73.1, 68.22 (68.15), 49.2 ($TEAB^+$), 32.8 (acetone), 10.8 ($TEAB^+$) (Figure S12). **ESI-HRMS** Exact mass (443.0651), Observed mass (443.0708) (Figure S16A).

^{1}H NMR of **diastereomer 2** (400 MHz, 10% D_2O in H_2O) δ 7.86 (s, 1H), 6.72 (s, 1H), 6.38 (s, 1H), 5.82 (d, J = 5.2 Hz, 1H), 4.41-4.39 (m, 1H), 4.24-4.23 (m, 1H), 4.10-4.07 (m, 2H) (Figure S13). ^{31}P NMR of **diastereomer 2** (162 MHz, 10% D_2O in H_2O) δ 46.70 (Figure S14). ^{13}C NMR of **diastereomer 2** (126 MHz, 10% D_2O in H_2O) δ 164.8, 158.6, 151.6, 151.0, 136.1, 123.37 (123.28), 117.1, 116.01 (115.95), 86.6, 83.22 (83.14), 73.6, 70.6, 65.69 (65.63), 46.5 ($TEAB^+$), 8.3 ($TEAB^+$) (Figure S15). **ESI-HRMS** Exact mass (443.0651), Observed mass (443.0663) (Figure S16B).

Synthesis of bridged GC dinucleotides (Gp*pC and Gps*pC)

Native 2-aminoimidazolium bridged dinucleotide (Gp*pC) was synthesized as previously reported from two monomers, cytidine 5′-phosphoryl-(1-hydroxy-7-azabenzotriazole) (CpOAt) and guanosine 5′-phosphoryl-(2-aminoimidazole) (*pG).[4]

The phosphorothioate version of the 2-aminoimidazolium bridged GC dinucleotide (Gps*pC) was synthesized similarly from the diastereomerically pure activated phosphorothioate monomer (*psG) and cytidine 5′-phosphoryl-(1-hydroxy-7-azabenzotriazole) (CpOAt). Excess CpOAt was applied to improve the conversion rate of phosphorothioate monomer. 10 μmol diastereomerically pure *psG and 40 μmol CpOAt were dissolved in deionized water (2 mL) and adjusted pH to 7.5 with NaOH/HCl, then stirred for 2 hours at room temperature. After the reaction was completed, the mixture was purified by reverse phase HPLC using a C18 column with (A) 10 mM TEAB buffer (pH 8.0) and (B) acetonitrile. The sample was eluted between 3% and 8% B over 10 CVs with a flow rate of 20 mL/min. Fractions containing product were adjusted to pH 7.0 and lyophilized at -20 °C. As we did not obtain enough material for NMR analysis, the product identity was confirmed by high-resolution mass spectrometry using an ESI Agilent 6520 Q-TOF LC-MS (Figure S17).

Synthesis of oligonucleotides

Oligonucleotides for crystallization were synthesized on either an Expedite 8909 DNA/RNA synthesizer or a K&A H-6 Oligo Synthesizer, using phosphoramidites from ChemGenes (Wilmington, MA) and reagents from Glen Research (Sterling, VA). Oligonucleotides were cleaved from the CPG solid support and deprotected with 3:1 ammonium hydroxide / ethanol at 55 °C for 16 hours. The mixtures were lyophilized, and the 2′-TBDMS protecting group was removed by incubation with 56% triethylamine trihydrofluoride in dimethyl sulfoxide at 65 °C for 2.5 hours. Oligonucleotides were purified by PAGE, and purity was confirmed by Agilent 6520 Q-TOF LC-MS.

The primer and template oligonucleotides used for nonenzymatic primer extension studies were purchased from Integrated DNA Technologies (Coralville, IA).

Nonenzymatic primer extension reactions

All reactions used the primer 5′-/Cy3/GCG UAG ACU GAC UG-3′. The template RNA sequence was 5′-AAA GCC AGU CAG UCU ACG C-3′. The primer/template complexes were prepared in an annealing buffer with 17 μM primer, 25.5 μM template, 1 M Tris-Cl pH 8.0. The solution was heated at 90 °C for 1 min and then slowly cooled to 25 °C at a rate of 0.1 °C/s in a thermal cycler.

For the primer extension reactions for Mg^{2+} binding kinetics, the diastereomerically pure *psG monomers were prepared as a 100 mM stock solution and CpOAt was prepared as a 200 mM stock

solution. The *psG and CpOAt were pre-mixed at 2:1 ratio for 10 min. In a 0.2 mL PCR tube, the primer/template reaction solution and $MgCl_2$ solution were placed at the bottom, while the mixed activated monomers were placed in the lid or on the wall, followed by immediate spin down to mix the solution and initiate the reaction. The final reaction solution containing 1.7 μM primer, 2.6 μM template, 100 mM Tris-Cl pH 8.0, 20 mM *psG and 20 mM CpOAt with specified concentrations of $MgCl_2$. At each time point, 1 μL of reaction sample was added to 9 μL of a quenching buffer containing 100 mM EDTA and 80% formamide.

For the primer extension reactions with different metal ions, the native Gp*pC and diastereomerically pure Gps*pC dinucleotides were prepared as 20 mM stock solutions. $MgCl_2$, $MnCl_2$ and $CdCl_2$ were prepared as 250 mM stock solutions. The final reaction mixtures contained 1.7 μM primer, 2.6 μM template, 50 mM $MgCl_2$, $MnCl_2$ or $CdCl_2$, 100 mM Tris-Cl pH 8.0 and 10 mM Gp*pC or Gps*pC.

Urea–PAGE analysis

Primer extension products were resolved by 20% (19:1) polyacrylamide gel electrophoresis (PAGE) with 7 M urea, in 1× TBE gel running buffer. The gels were scanned with an Amersham Typhoon RGB Biomolecular Imager (GE Healthcare Life Sciences). The fluorescently labeled primer and extended products were visualized, and then quantified using ImageQuant TL software to obtain relative band intensities. Rate constants were determined by fitting the fraction of remaining primer vs. time to a log-linear decay curve.

Crystallization

The 14-mer oligonucleotide 5′-*CCC G*AC UUA AGU CG-3′ and 13-mer oligonucleotide 5′-*CCC G*AC UUA AGU C-3′ were used for product preparation and crystallization. *C* denotes LNA 5-methylcytidine residues and *G* denotes LNA guanosine residues, while A, C, G and U denote unmodified RNA residues. For Mg^{2+} catalyzed primer extension products with pure diastereomeric monomers, 0.5 mM primer was incubated with 20 mM diastereomerically pure *psG and 50 mM $MgCl_2$ at room temperature for 72 hours. The product mixture was then used directly for crystallization screening without further purification. For the primer extension products with mixed diastereomeric monomers, the reaction was conducted with 50 μM primer, 5 mM racemic *psG monomer, 5 mM $MgCl_2$ or $CdCl_2$, 100 mM Tris-HCl pH 7.0 at room temperature for 72 hours. The products were then purified by reverse phase analytical HPLC over a C18 column. The purified product was dissolved to 1 mM in nuclease-free water and mixed with 20 mM dGMP at 1:1 ratio. The sample was heated at 90 °C for 1 min and then slowly cooled to 25 °C at a rate of 0.1 °C/s in a thermal cycler machine.

Crystal Screen HT, Index HT, Natrix HT (Hampton Research, Aliso Viejo, CA) and Nuc-Pro HTS (Jena Bioscience, Jena, Germany) were used to screen crystallization conditions at 20 °C

using the sitting-drop vapor diffusion method. An NT8 robotic system and Rock Imager (Formulatrix, Waltham, MA) were used for crystallization screening and monitoring the crystallization process. Optimal crystallization conditions are listed in the Supplementary Table S2.

Crystal data collection, structure determination and refinement

Diffraction data were collected at a wavelength of about 1 Å (exact values are in Table S2) under a liquid nitrogen stream at 99 K on Beamline 201, 503 or 822 at the Advanced Light Source in the Lawrence Berkeley National Laboratory (USA). The crystals were exposed for 0.25 s per image with a 0.25 Å oscillation angle. The distances between detector and the crystal were set to 180–300 mm. The data were processed by HKL2000.[5] The structures were solved by molecular replacement by PHASER[6] using structure of 5L00 as the searching model.[7] All structures were refined by Refmac5 in CCP4i. After several cycles of refinement, well resolved water molecules and metal atoms were added in Coot.[8] Data collection, phasing, and refinement statistics of the determined structures are listed in Supplementary Tables S3 and S4.

Supplementary Figures and Tables

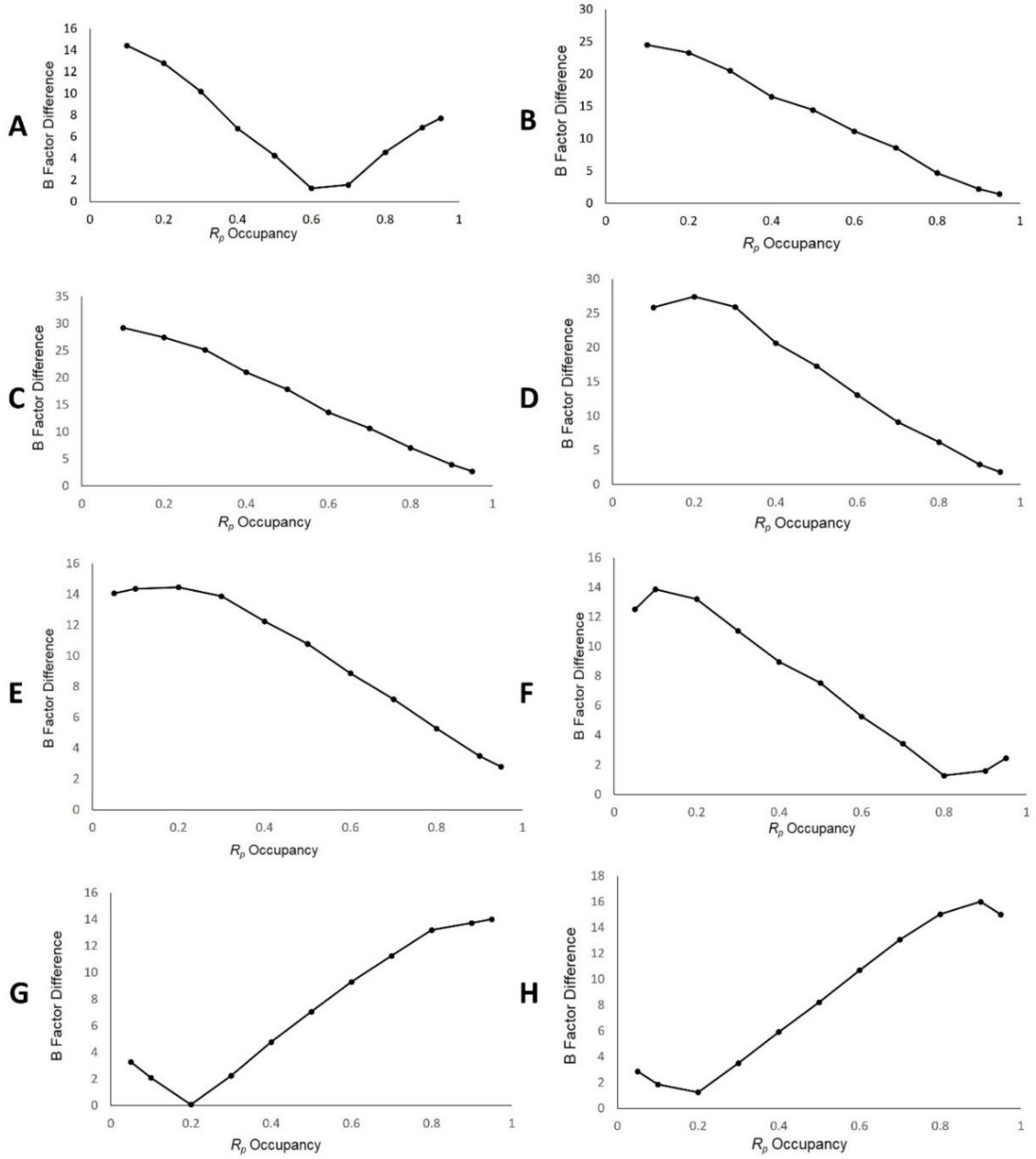

Figure S1. B factor constancy validation for +1 product chirality. (A) A chain of the product of Mg^{2+} catalyzed primer extension with diastereomer 1 monomer (Figure 3C, PDB:7U89); (B) B chain of the product of Mg^{2+} catalyzed primer extension with diastereomer 1 monomer (Figure 3C, PDB:7U89); (C) A chain of the product of Mg^{2+} catalyzed primer extension with diastereomer 2 monomer (Figure 3D, PDB:7U8A); (D) B chain of the product of Mg^{2+} catalyzed primer extension with diastereomer 2 monomer (Figure 3D, PDB:7U8A); (E) A chain of the product of Mg^{2+} catalyzed primer extension with mixed monomer diastereomers (Figure 4A, PDB:8VAW); (F) B chain of the product of Mg^{2+} catalyzed primer extension with mixed monomer diastereomers (Figure 4A, PDB: 8VAW); (G) A chain of the product of Cd^{2+} catalyzed primer extension with mixed monomer diastereomers (Figure 4B, PDB: 8VAX); (H) B chain of the product of Cd^{2+} catalyzed primer extension with mixed monomer diastereomers (Figure 4B, PDB: 8VAX).

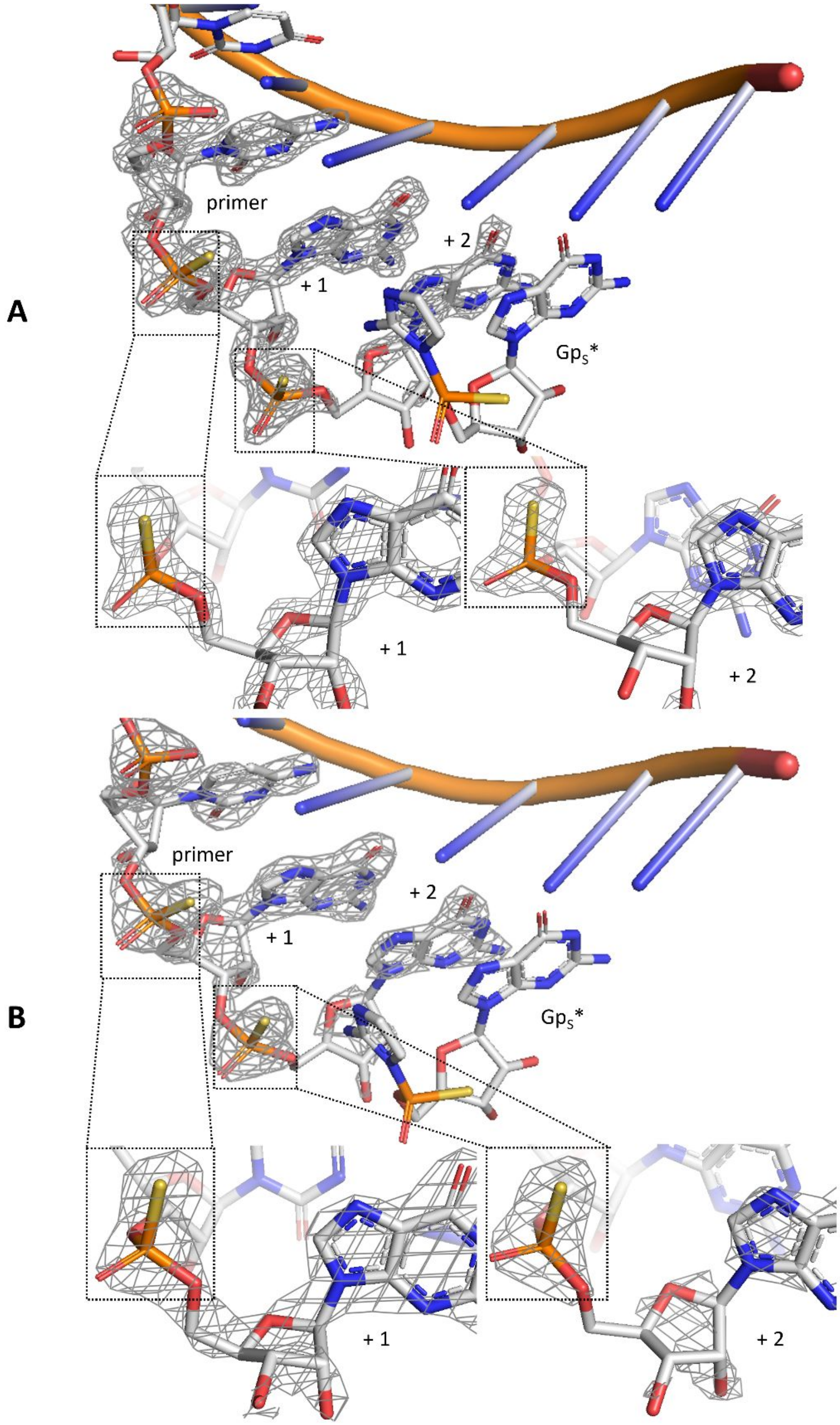

Figure S2. Structure of the products of Mg^{2+} catalyzed 13mer primer extension with two diastereomer monomers. (A) Product structure of the Mg^{2+} catalyzed 13-mer primer extension with diastereomer 1 (PDB: 7U87). (B) Product structure of the Mg^{2+} catalyzed 13-mer primer extension with diastereomer 2 (PDB: 7U88). (Meshes indicate the composite omit $2DF_o$-mF_c maps contoured at 2.0 σ.)

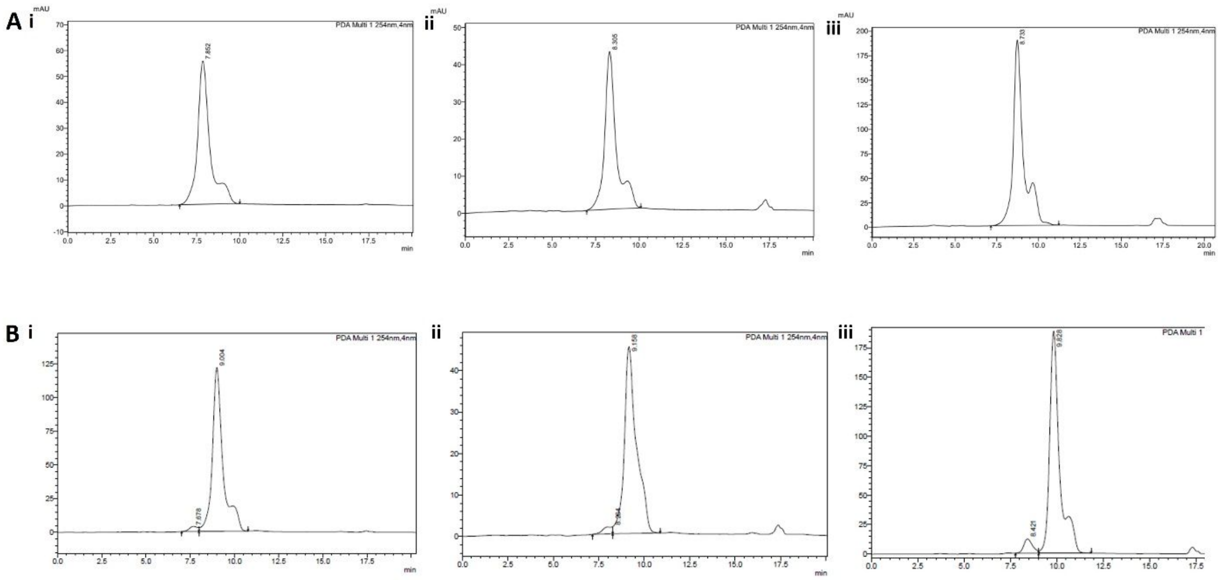

Figure S3. HPLC analysis of the purity of the phosphorothioate monomer diastereomers (retention time for D1 is 7.5-8.5 min, for D2 is 9-10 min, bridged dinucleotide is 17-17.5 min). (A) Purity of diastereomer 1 (i) immediately after HPLC purification (ii) after lyophilizing and redissolving (iii) after several freeze-thaw cycles. (B) Purity of diastereomer 2 (i) immediately after HPLC purification (ii) after lyophilizing and redissolving (iii) after several freeze-thaw cycles.

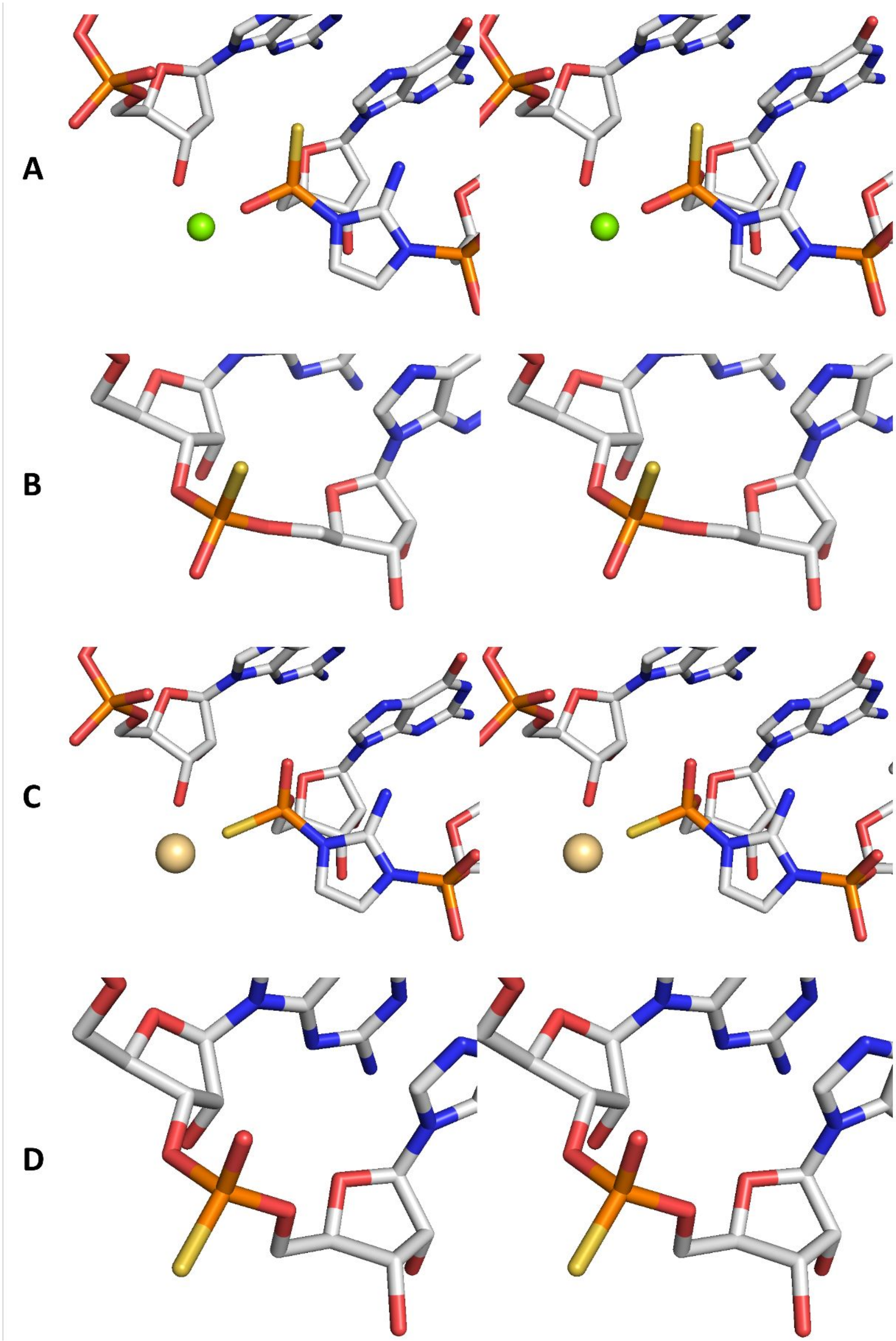

Figure S4. Parallel-eye stereo pairs for Figure 5. (A) Mg^{2+} coordinates to the lower oxygen on S_P-substrate. (B) Mg^{2+} catalysis leads to R_P-product. (C) Cd^{2+} coordinates to the lower sulfur on R_P-substrate. (D) Cd^{2+} catalysis leads to S_P-product.

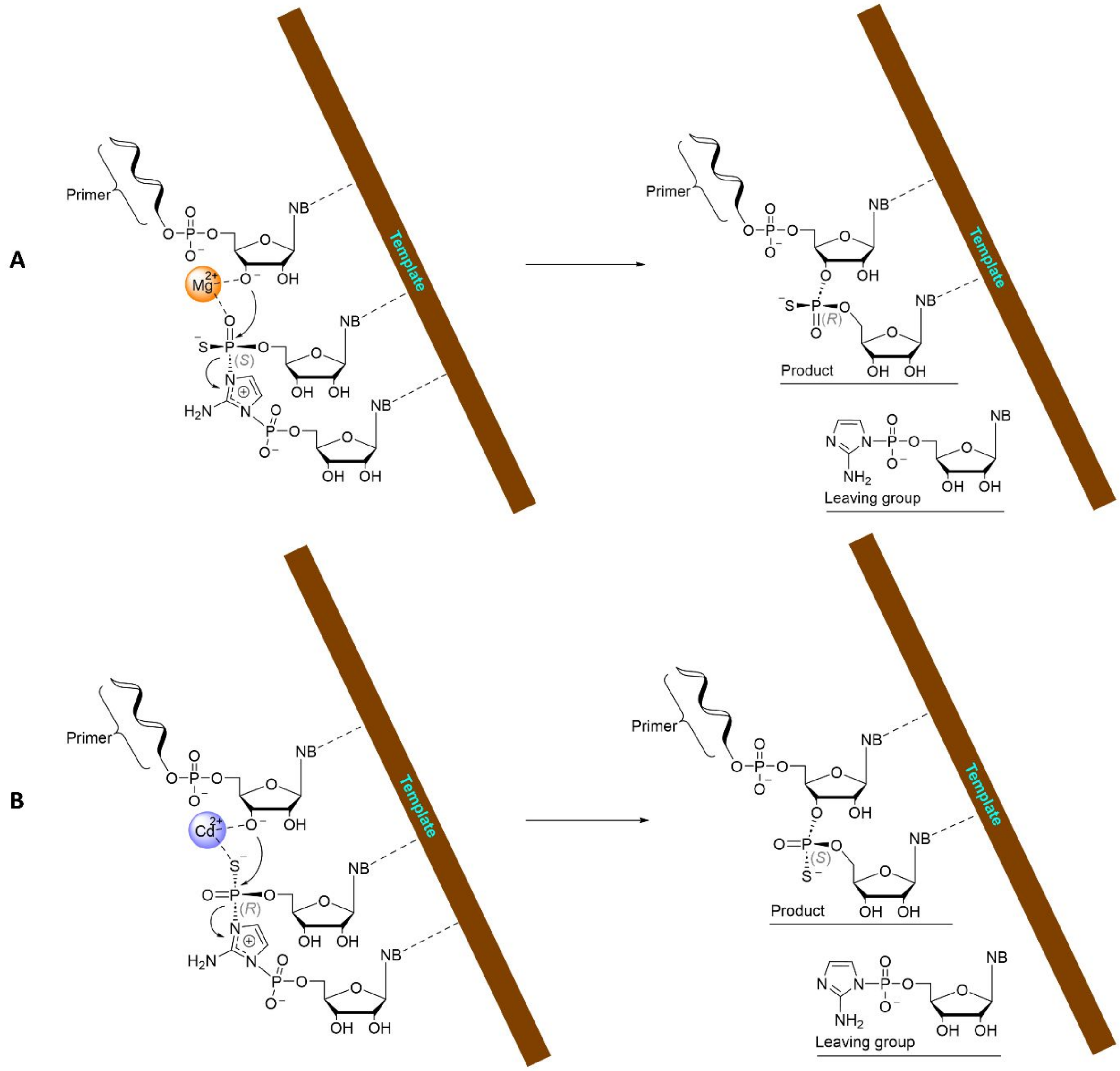

Figure S5. Arrow-pushing mechanisms for (A) Mg^{2+} catalyzed primer extension and (B) Cd^{2+} catalyzed primer extension with phosphorothioate substrates.

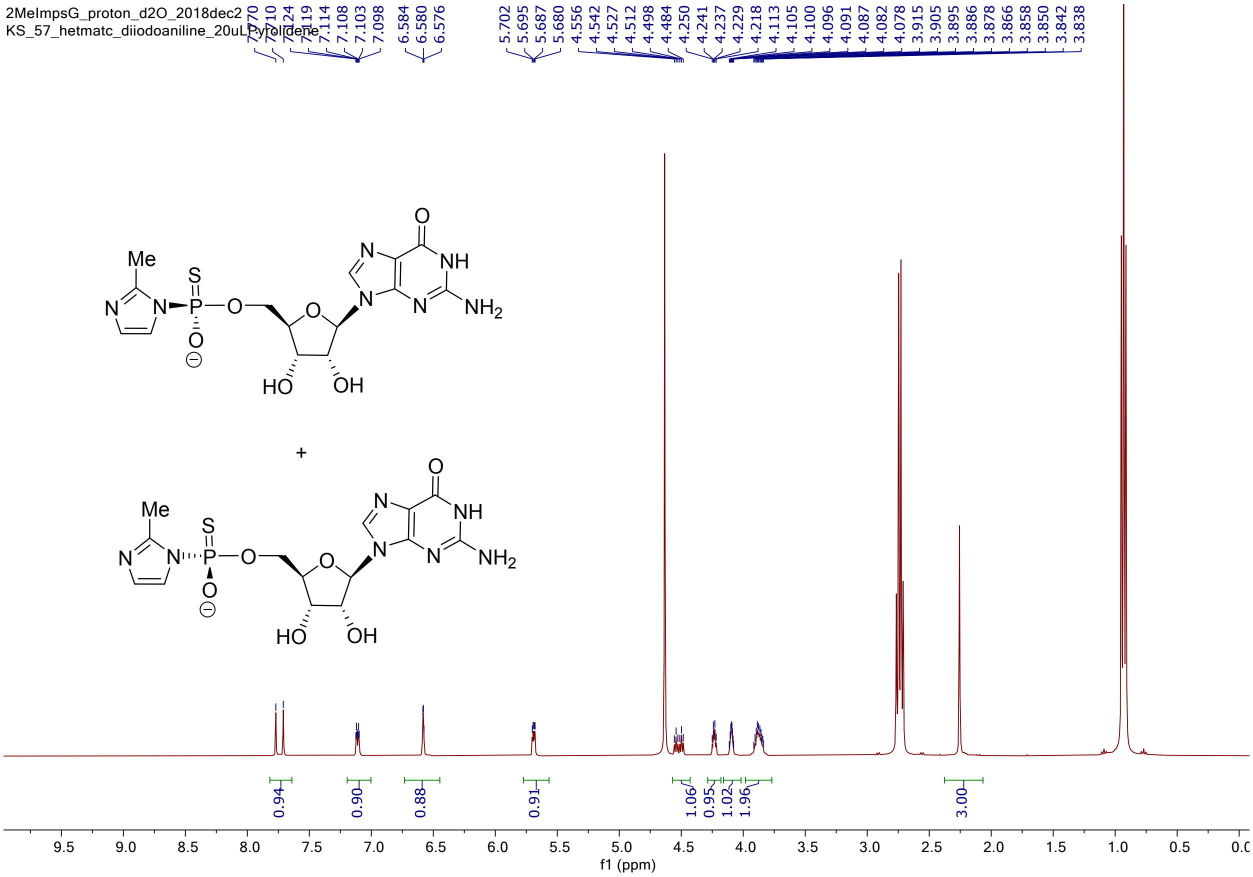

Figure S6. ^{1}H NMR spectrum of compound **S3.** (400 MHz, D_2O) δ 7.77 and 7.71 (s, 1H), 7.12-7.10 (m, 1H), 6.58-6.5 (m, 1H), 5.70-5.68 (m, 1H), 4.54 and 4.50 (t, J = 5.6 Hz, 1H), 4.25-4.22 (m, 1H), 4.11-4.08 (m, 1H), 3.92-3.84 (m, 1H).

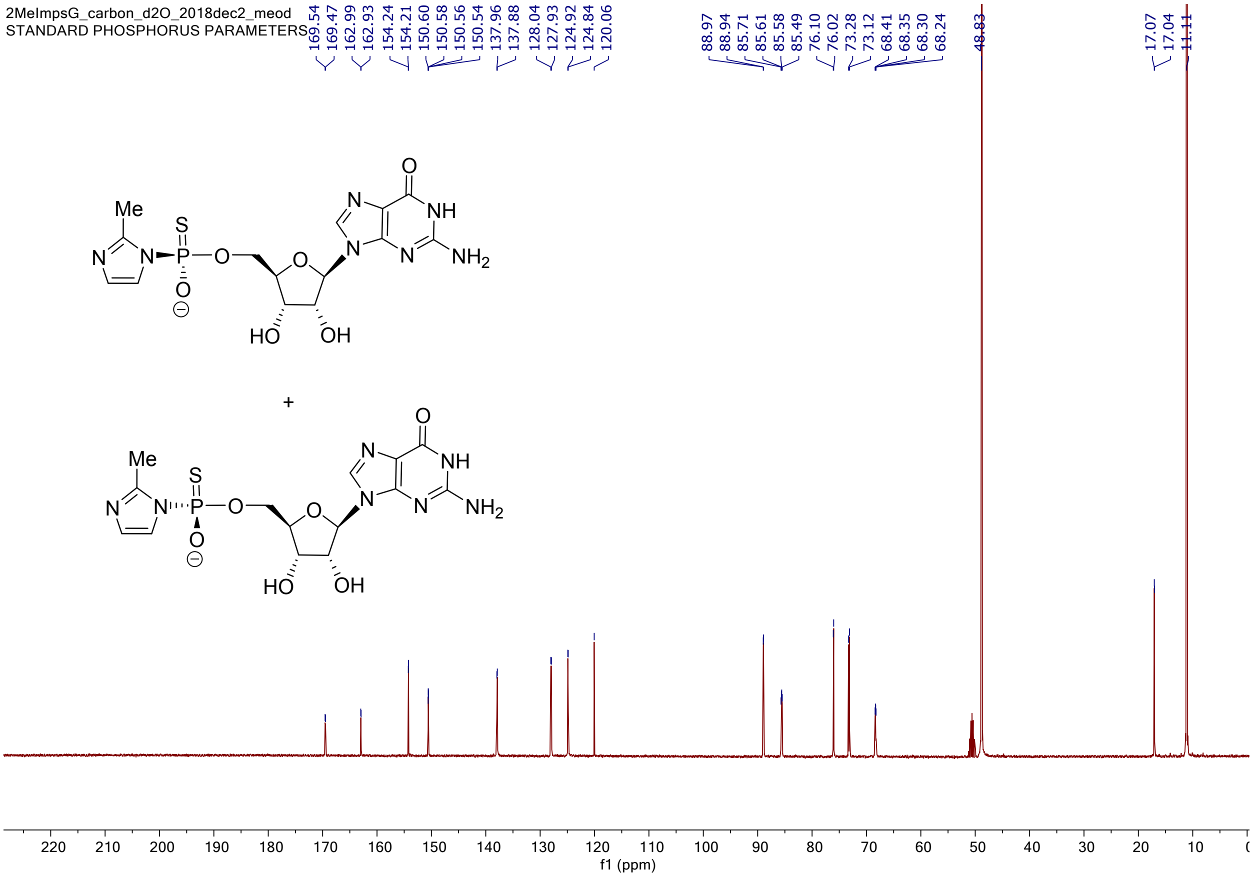

Figure S7. ^{13}C NMR spectrum of compound **S3.** **(**101 MHz, D_2O**)** δ 169.54 (169.47), 162.99 (162.93), 154.24 (154.21), 150.60 (150.58, 150.56, 150.54), 137.96 (137.88), 128.04 (127.93), 124.92 (124.84), 120.1, 88.97 (88.94), 85.71 (85.61, 85.58, 85.49), 76.10 (76.02), 73.28 (73.12), 68.41 (68.35, 68.30, 68.24), 48.8 ($TEAB^+$), 17.07 (17.04), 11.1.

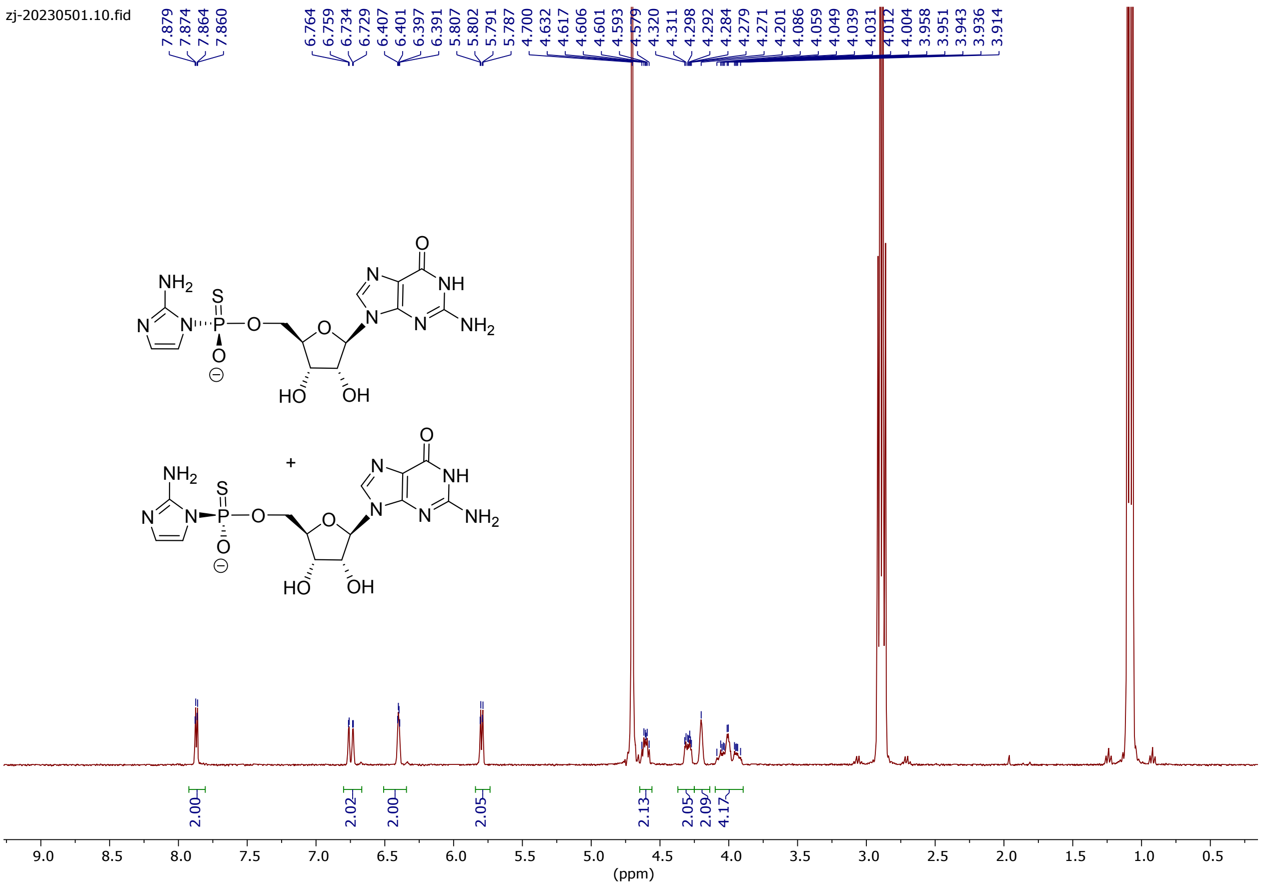

Figure S8. ^{1}H NMR spectrum of compound **S4** (diastereomeric mixture). (400 MHz, D_2O) δ 7.88-7.86 (m, 2H), 7.76-7.73 (m, 2H), 6.41-6.39 (m, 2H), 5.81-5.79 (m, 2H), 4.63-4.58 (m, 2H), 4.32-4.27 (m, 2H), 4.21 (s, 2H), 4.09-3.91 (m, 4H).

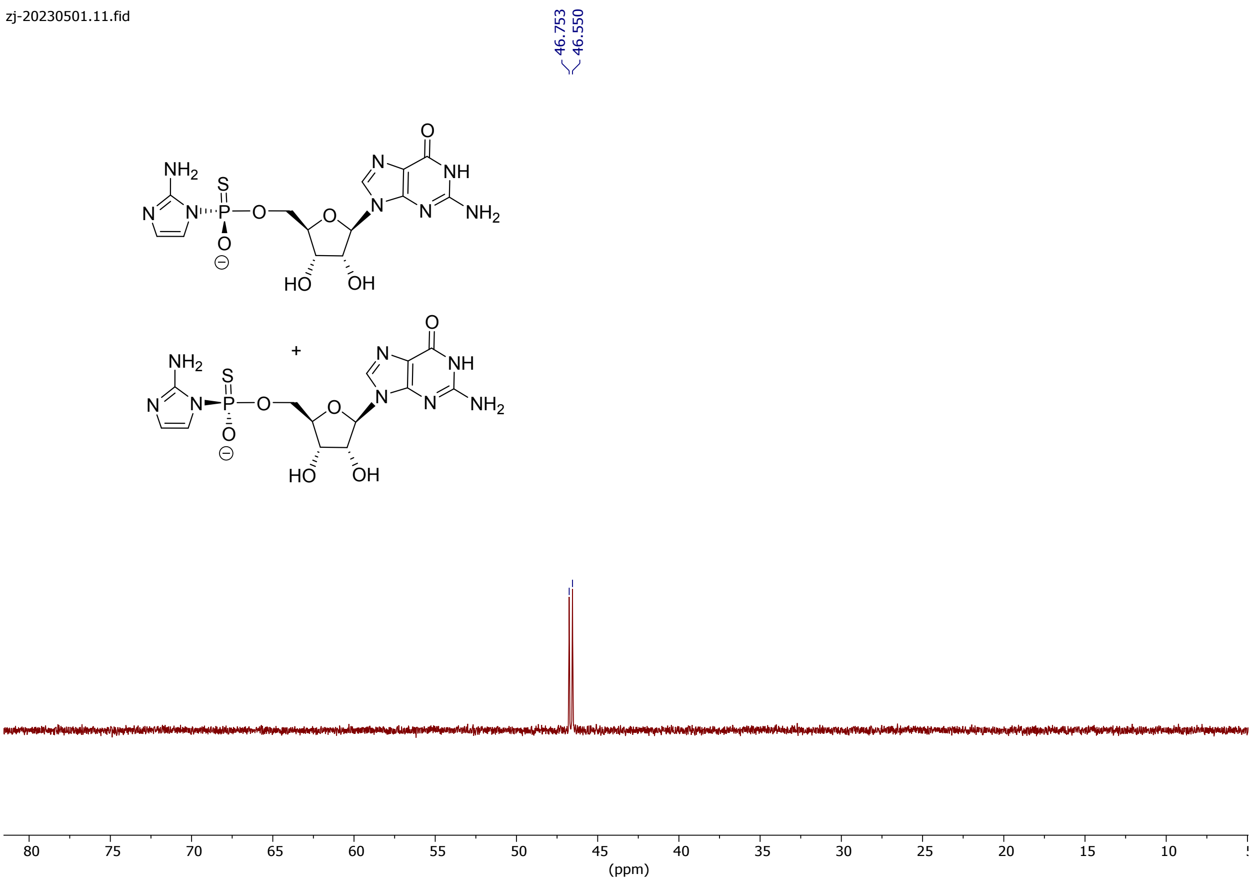

Figure S9. ^{31}P NMR spectrum of compound **S4** (diastereomeric mixture). (162 MHz, D_2O) δ 46.75, 46.55.

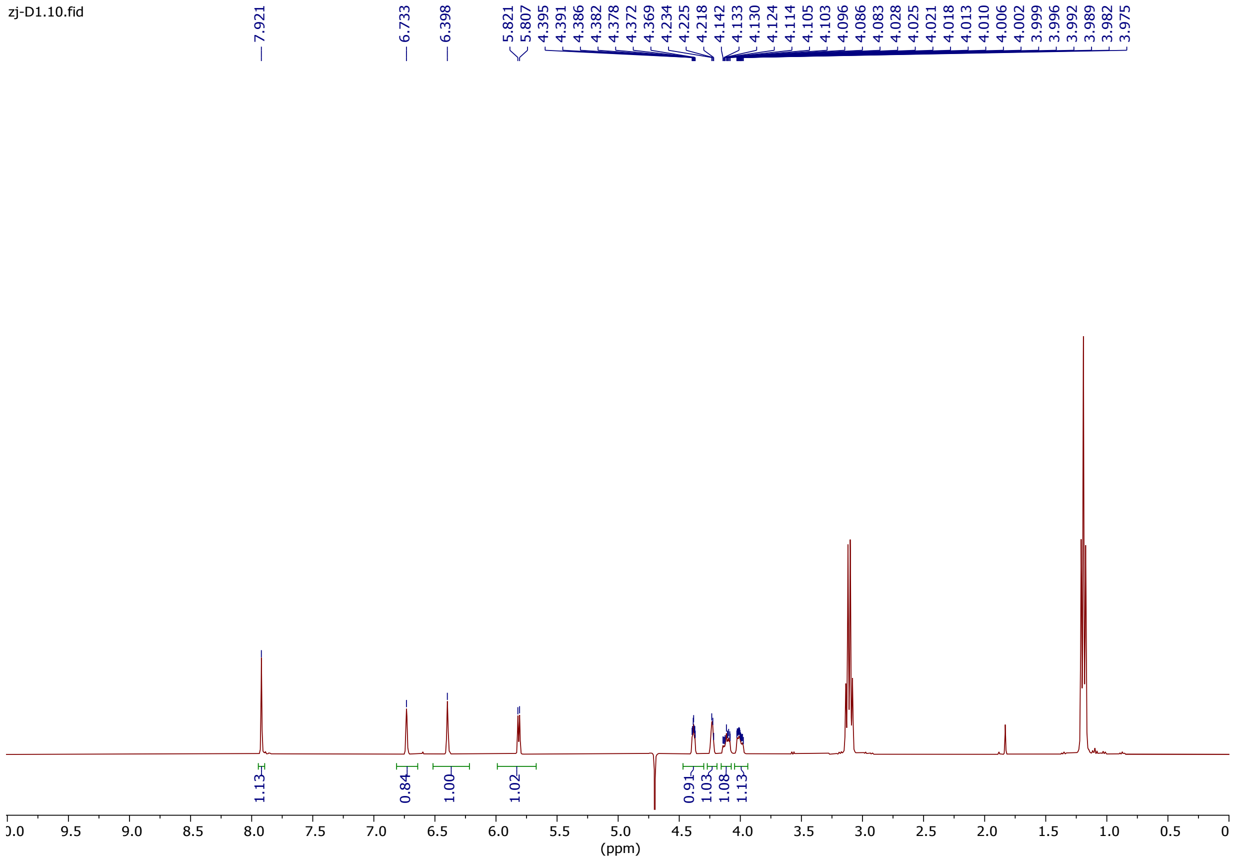

Figure S10. ^{1}H NMR spectrum of diastereomer 1. (400 MHz, 10% D_2O in H_2O) δ 7.92 (s, 1H), 6.73 (s, 1H), 6.40 (s, 1H), 5.82 (d, *J* = 5.6 Hz, 1H), 4.40-4.37 (m, 1H), 4.23-4.22 (m, 1H), 4.14-4.08 (m, 2H), 4.03-3.98 (m, 1H).

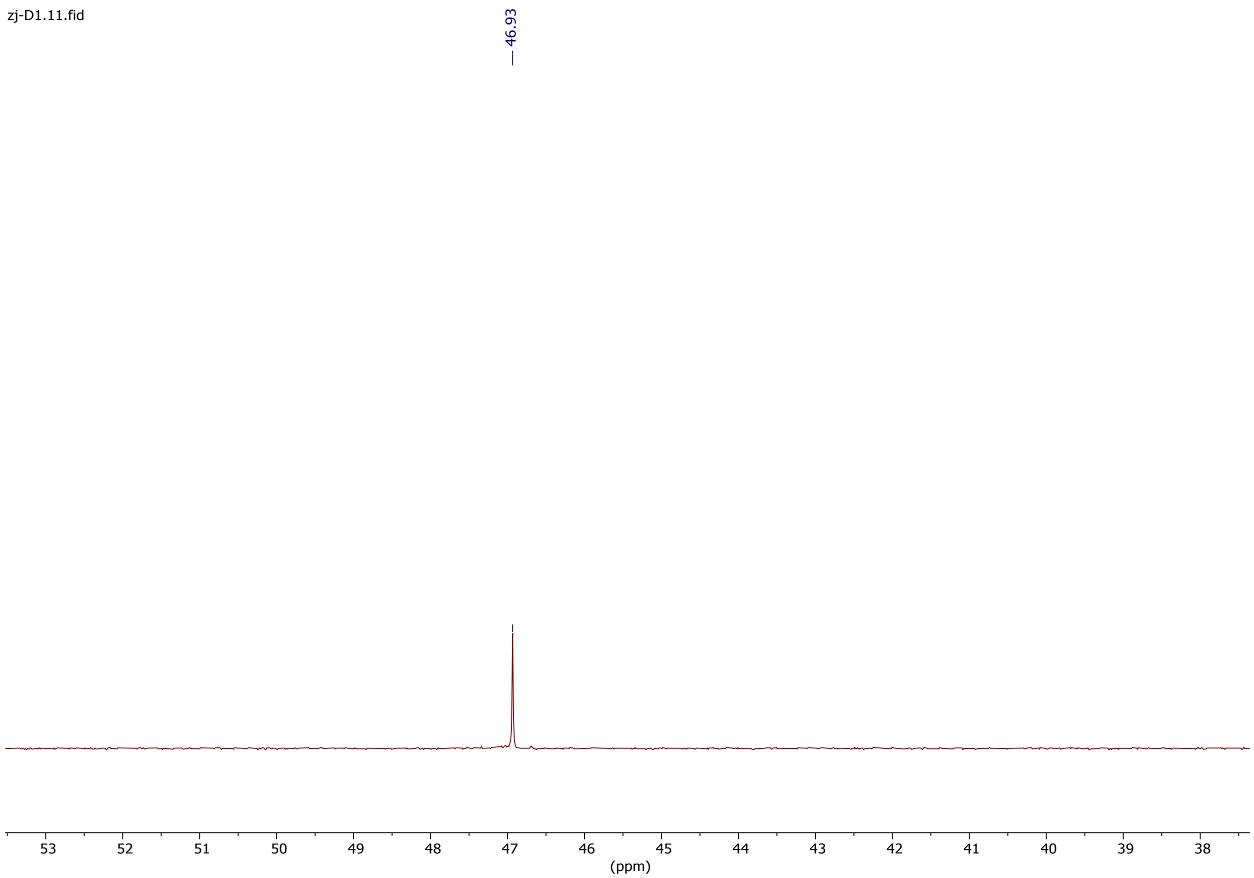

Figure S11. ^{31}P NMR spectrum of diastereomer 1. (162 MHz, 10% D_2O in H_2O) δ 46.93.

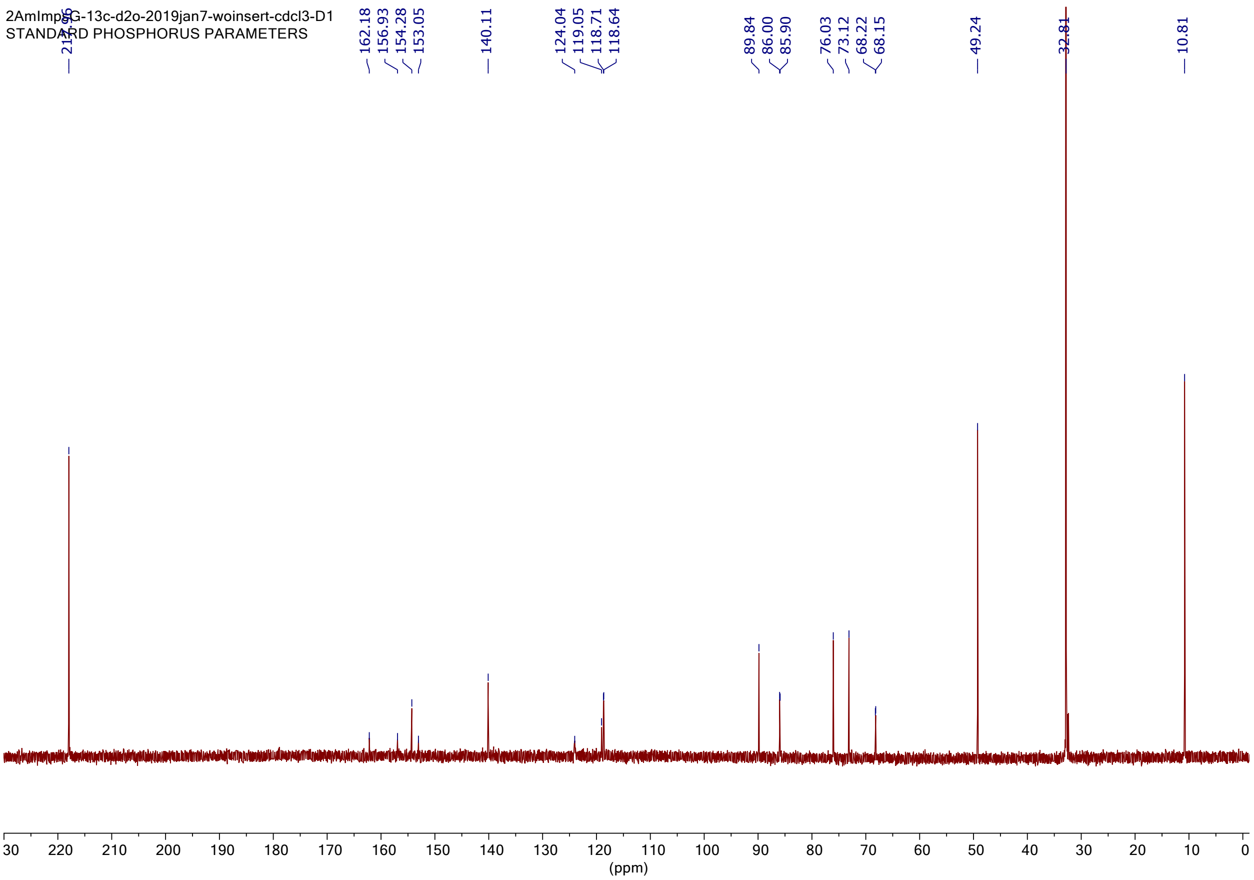

Figure S12. ^{13}C NMR spectrum of diastereomer 1. (101 MHz, D_2O) δ 218.0 (acetone), 162.2, 156.9, 154.3, 153.1, 140.1, 124.0, 119.1, 118.71 (118.64), 89.8, 86.00 (85.90), 76.0, 73.1, 68.22 (68.15), 49.2 ($TEAB^+$), 32.8 (acetone), 10.8 ($TEAB^+$).

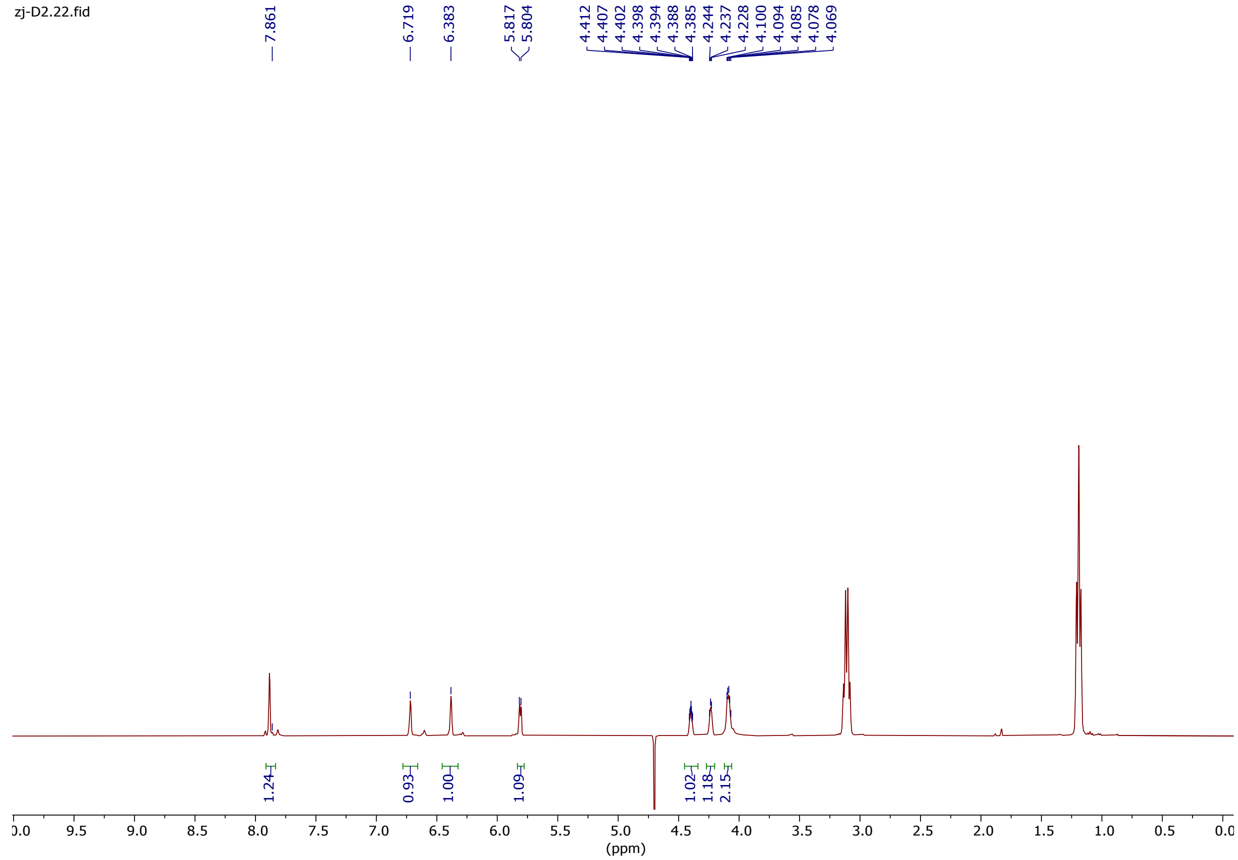

Figure S13. ^{1}H NMR spectrum of diastereomer 2. (400 MHz, 10% D_2O in H_2O) δ 7.86 (s, 1H), 6.72 (s, 1H), 6.38 (s, 1H), 5.82 (d, J = 5.2 Hz, 1H), 4.41-4.39 (m, 1H), 4.24-4.23 (m, 1H), 4.10-4.07 (m, 2H).

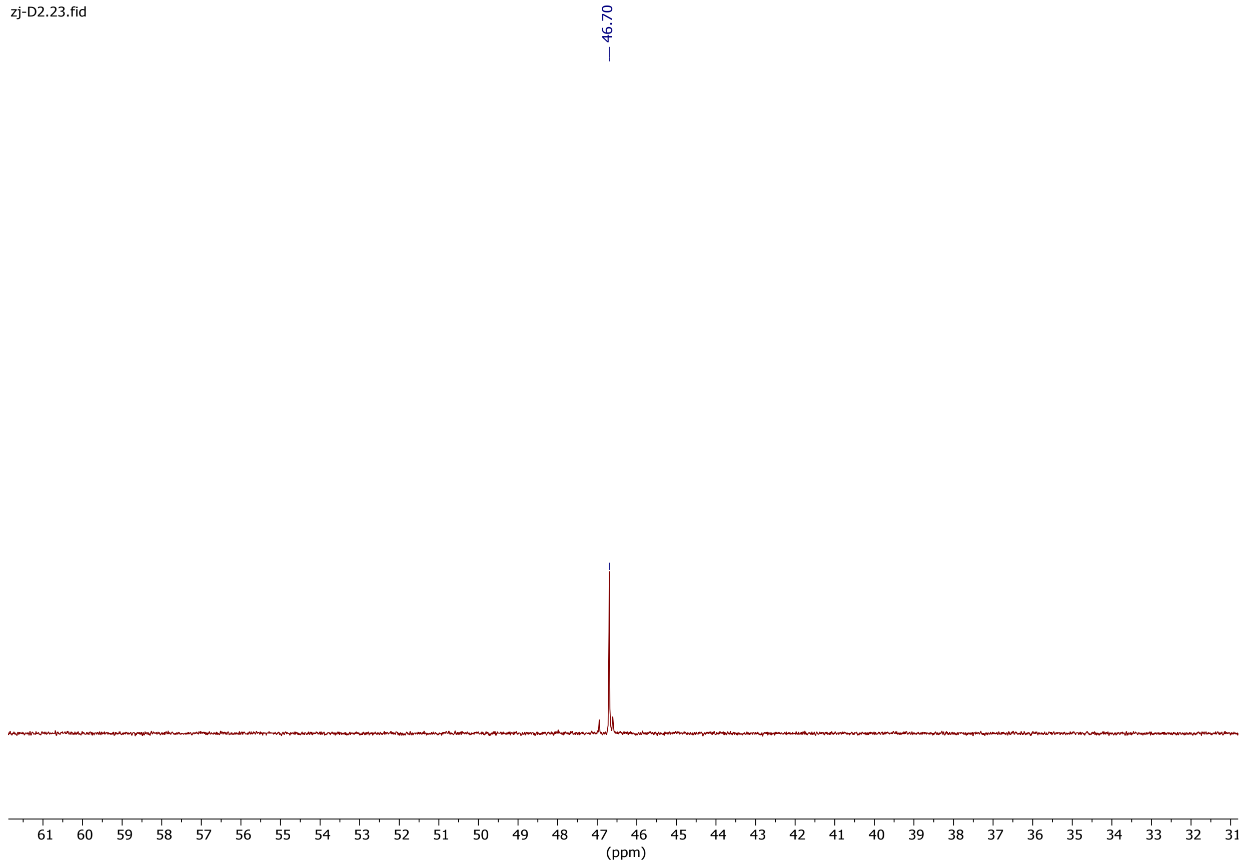

Figure S14. ^{31}P NMR spectrum of diastereomer 2. (162 MHz, 10% D_2O in H_2O) δ 46.70.

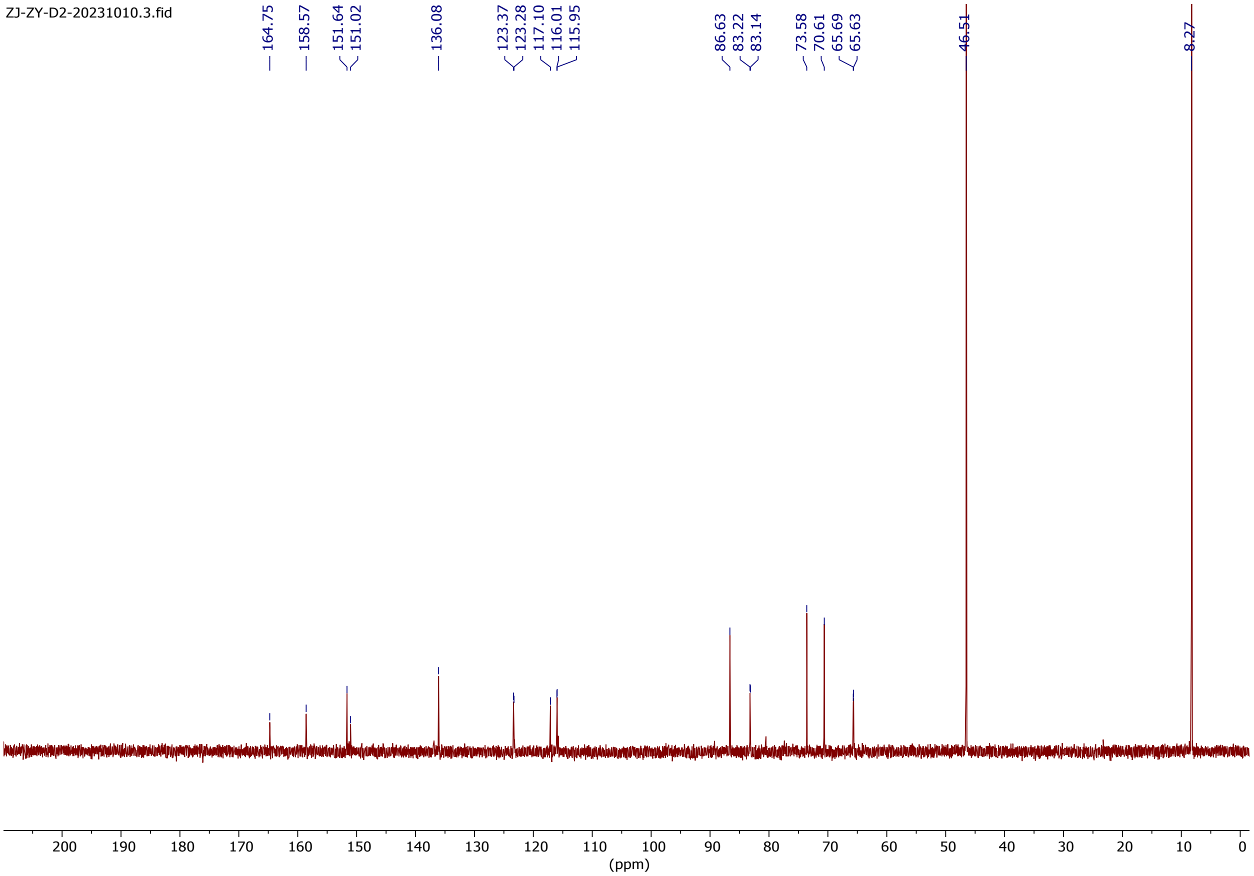

Figure S15. ^{13}C NMR spectrum of diastereomer 2. (126 MHz, 10% D_2O in H_2O) δ 164.8, 158.6, 151.6, 151.0, 136.1, 123.37 (123.28), 117.1, 116.01 (115.95), 86.6, 83.22 (83.14), 73.6, 70.6, 65.69 (65.63), 46.5 ($TEAB^+$), 8.3 ($TEAB^+$).

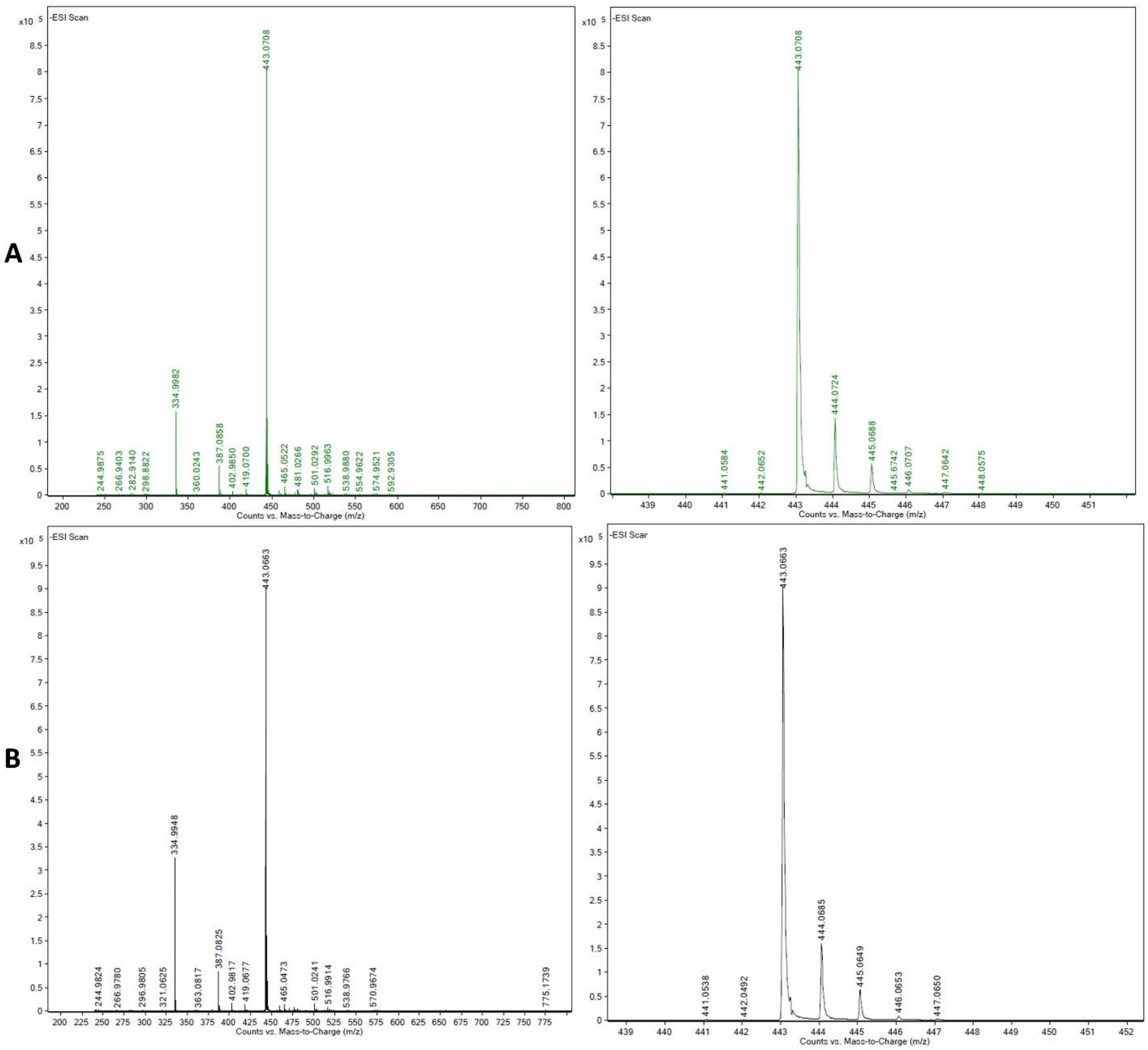

Figure S16. High-resolution mass spectrometry for thiophosphoro-2-aminoimidazolides (*psG, chemical formula: $C_{13}H_{17}N_8O_6PS$), calculated monoisotopic mass $[M\text{-}H]^-$ = 443.0651. (A) diatereomer 1, determined mass $[M\text{-}H]^-$ = 443.0708; (B) diastereomer 2, determined mass $[M\text{-}H]^-$ = 443.0663.

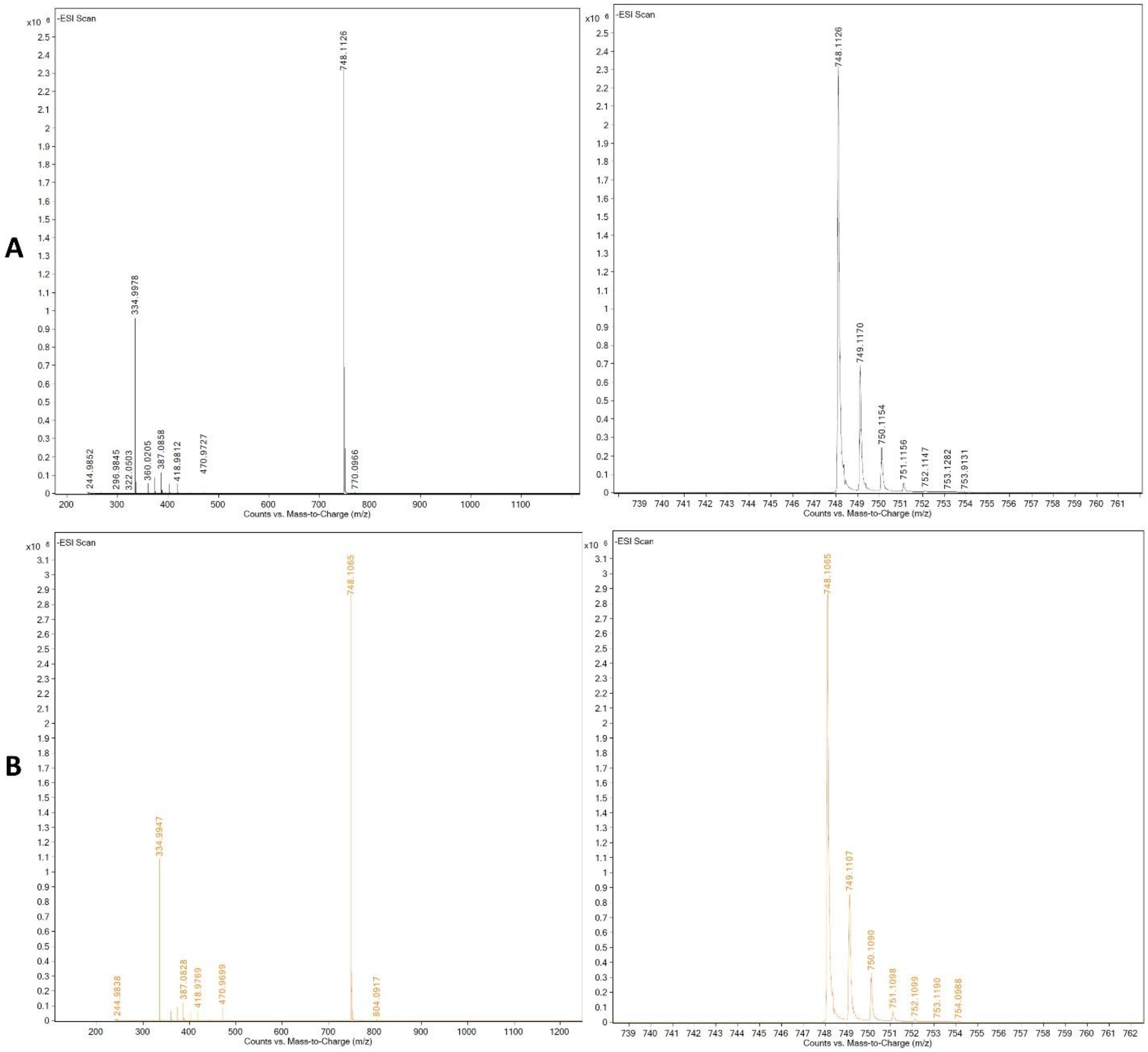

Figure S17. High-resolution mass spectrometry for phosphorothioate bridged GC dinucleotides (Gps*pC, chemical formula: $C_{22}H_{29}N_{11}O_{13}P_2S$), calculated monoisotopic mass [M-H]$^-$ = 748.1064. (A) diastereomer 1, determined mass [M-H]$^-$ = 748.1126; (B) diastereomer 2, determined mass [M-H]$^-$ = 748.1065.

Table S1. Phenix occupancy refinement results.

PDB code	Primer	Metal	Monomer	Chain A R_P: S_P	Chain B R_P: S_P
7U89	14-mer	Mg	Diastereomer 1	0.97 : 0.03	1.00 : 0.00
7U8A	14-mer	Mg	Diastereomer 2	0.99 : 0.01	1.00 : 0.00
8VAW	14-mer	Mg	Mixture of 1 and 2	1.00 : 0.00	1.00 : 0.00
8VAX	14-mer	Cd	Mixture of 1 and 2	0.06 : 0.94	0.00 : 1.00

Table S2. Optimized Conditions for Crystallization.

Entry	Content	Condition
1	Product of 14-mer primer, diastereomer 1 monomer and magnesium	20 mM Magnesium chloride hexahydrate, 50 mM MOPS pH 7.0, 2.0 M Ammonium sulfate, 0.5 mM Spermine.
2	Product of 14-mer primer, diastereomer 2 monomer and magnesium	26% v/v (+/-)-2-Methyl-2,4-pentanediol, 50 mM PIPES pH 7.0, 65 mM Magnesium chloride, 1 mM Cobalt (III) Hexamine chloride.
3	Product of 14-mer primer, mixed diastereomer monomers and magnesium	2.5 M Sodium chloride, 50 mM TRIS pH 7.5, 0.25 M Magnesium chloride.
4	Product of 14-mer primer, mixed diastereomer monomers and cadmium	35% w/v 1,6-Hexanediol, 50 mM TRIS pH 8.5 75 mM Magnesium sulfate.
5	Product of 13-mer primer, diastereomer 1 monomer and magnesium	0.2 M Ammonium acetate, 0.1 M BIS-TRIS pH 6.5, 45% v/v (+/-)-2-Methyl-2,4-pentanediol.
6	Product of 13-mer primer, diastereomer 1 monomer and magnesium	1.5 M Lithium sulfate, 50 mM TRIS pH 8.5, 5 % w/v Glycerol.

Table S3. Parameters for X-ray data collection.

Entry	1	2	3	4	5	6
PDB code	7U89	7U8A	8VAW	8VAX	7U87	7U88
Wavelength (Å)	1.00000	1.00000	0.97648	1.03579	1.00000	1.00000
Space group	P321	P321	P321	P321	P321	P321
Unit cell parameters (Å, ◦)	43.20, 43.20, 85.64, 90, 90, 120	42.87, 42.87, 82.76, 90, 90, 120	43.57, 43.57, 84.34, 90, 90, 120	43.47, 43.47, 83.89, 90, 90, 120	42.74, 42.74, 83.28, 90, 90, 120	42.72, 42.72, 85.82, 90, 90, 120
Resolution range (Å)	50 - 1.65 (1.68 - 1.65)	50 - 2.10 (2.14 - 2.10)	50 - 1.25 (1.27 - 1.25)	50 - 1.42 (1.44 - 1.42)	50 - 1.70 (1.73 - 1.70)	50 - 2.14 (2.18 - 2.14)
Unique reflections	11662 (553)	5553 (273)	26004 (1069)	16509 (502)	10188 (474)	5344 (229)
Completeness (%)	99.6 (98.9)	99.9 (100)	98.4 (82.0)	91.5 (56.4)	99.9 (98.8)	99.2 (88.8)
Redundancy	10.1 (7.8)	10.2 (9.9)	8.4 (3.5)	7.2 (2.4)	9.9 (6.5)	9.5 (5.7)
R_{merge}(%)	8.6 (26.0)	7.9 (52.7)	3.9 (42.4)	9.5 (28.6)	8.9 (34.8)	7.7 (40.2)
$\langle I/\sigma(I) \rangle$	21.0 (5.6)	25.8 (4.2)	50.2 (2.9)	19.6 (3.4)	19.4 (2.7)	24.2 (2.2)

Table S4. Parameters for structure refinement.

Entry	1	2	3	4	5	6
PDB code	7U89	7U8A	8VAW	8VAX	7U87	7U88
RNA strands per asymmetric unit	2	2	2	2	2	2
Resolution range (Å)	28.56 - 1.65	41.42 - 2.10	37.73 - 1.25	41.94 - 1.42	41.67 - 1.70	42.95 - 2.14
Number of reflections	9696	5254	23685	15502	8558	4411
R_{work} (%)	22.0	19.9	18.8	17.5	19.6	21.7
R_{free} (%)	28.2	28.2	19.1	21.8	24.0	29.2
Bond length R.M.S. (Å)	0.021	0.038	0.022	0.019	0.033	0.018
Bond angle R.M.S. (◦)	2.98	2.71	2.93	2.87	3.26	2.60
Average B-factors (Å^2)	15.20	23.63	13.78	12.69	15.89	22.40

References

(1) Afonine, P. V.; Grosse-Kunstleve, R. W.; Echols, N.; Headd, J. J.; Moriarty, N. W.; Mustyakimov, M.; Terwilliger, T. C.; Urzhumtsev, A.; Zwart, P. H.; Adams, P. D. Towards automated crystallographic structure refinement with phenix. refine. *Acta Crystallographica Section D: Biological Crystallography* **2012**, *68* (4), 352-367.

(2) Liebschner, D.; Afonine, P. V.; Baker, M. L.; Bunkóczi, G.; Chen, V. B.; Croll, T. I.; Hintze, B.; Hung, L.-W.; Jain, S.; McCoy, A. J. Macromolecular structure determination using X-rays, neutrons and electrons: recent developments in Phenix. *Acta Crystallographica Section D: Structural Biology* **2019**, *75* (10), 861-877.

(3) Yamasaki, K.; Akutsu, Y.; Yamasaki, T.; Miyagishi, M.; Kubota, T. Enhanced affinity of racemic phosphorothioate DNA with transcription factor SATB1 arising from diastereomer-specific hydrogen bonds and hydrophobic contacts. *Nucleic Acids Research* **2020**, *48* (8), 4551-4561.

(4) Ding, D.; Zhou, L.; Giurgiu, C.; Szostak, J. W. Kinetic explanations for the sequence biases observed in the nonenzymatic copying of RNA templates. *Nucleic Acids Research* **2022**, *50* (1), 35-45.

(5) Otwinowski, Z.; Minor, W. [20] Processing of X-ray diffraction data collected in oscillation mode. In *Methods in enzymology*, Vol. 276; Elsevier, 1997; pp 307-326.

(6) McCoy, A. J.; Grosse-Kunstleve, R. W.; Adams, P. D.; Winn, M. D.; Storoni, L. C.; Read, R. J. Phaser crystallographic software. *Journal of applied crystallography* **2007**, *40* (4), 658-674.

(7) Zhang, W.; Tam, C. P.; Wang, J.; Szostak, J. W. Unusual base-pairing interactions in monomer–template complexes. *ACS Central Science* **2016**, *2* (12), 916-926.

(8) Emsley, P.; Lohkamp, B.; Scott, W. G.; Cowtan, K. Features and development of Coot. *Acta Crystallographica Section D: Biological Crystallography* **2010**, *66* (4), 486-501.
